# The cloud-free global energy balance and inferred cloud radiative effects: an assessment based on direct observations and climate models

**DOI:** 10.1007/s00382-018-4413-y

**Published:** 2018-08-21

**Authors:** Martin Wild, Maria Z. Hakuba, Doris Folini, Patricia Dörig-Ott, Christoph Schär, Seiji Kato, Charles N. Long

**Affiliations:** 1 Institute for Atmospheric and Climate Science, ETH Zurich, 8001 Zurich, Switzerland; 2 Department of Atmospheric Sciences, Colorado State University, Ft Collins, CO 80523 USA; 3 NASA Jet Propulsion Laboratory, California Institute of Technology, Pasadena, CA 91109 USA; 4 NASA Langley Research Center, 21 Langley Boulevard, Hampton, VA 23681-2199 USA; 5 NOAA ESRL GMD/CIRES, Boulder, CO 80305 USA

## Abstract

In recent studies we quantified the global mean Earth energy balance based on direct observations from surface and space. Here we infer complementary reference estimates for its components specifically under cloud-free conditions. While the clear-sky fluxes at the top of atmosphere (TOA) are accurately known from satellite measurements, the corresponding fluxes at the Earth’s surface are not equally well established, as they cannot be directly measured from space. This is also evident in 38 global climate models from CMIP5, which are shown to greatly vary in their clear-sky surface radiation budgets. To better constrain the latter, we established new clear-sky reference climatologies of surface downward shortwave and longwave radiative fluxes from worldwide distributed Baseline Surface Radiation Network sites. 33 out of the 38 CMIP5 models overestimate the clear-sky downward shortwave reference climatologies, whereas both substantial overestimations and underestimations are found in the longwave counterparts in some of the models. From the bias structure of the CMIP5 models we infer best estimates for the global mean surface downward clear-sky shortwave and longwave radiation, at 247 and 314 Wm^−2^, respectively. With a global mean surface albedo of 13.5% and net shortwave clear-sky flux of 287 Wm^−2^ at the TOA this results in a global mean clear-sky surface and atmospheric shortwave absorption of 214 and 73 Wm^−2^, respectively. From the newly-established diagrams of the global energy balance under clear-sky and all-sky conditions, we quantify the cloud radiative effects not only at the TOA, but also within the atmosphere and at the surface.

## Introduction

The global energy balance fundamentally constrains the energy fluxes in the Earth’s climate system. Radiative transfer through the atmosphere can be modified by both cloud-related processes and processes within the cloud-free atmosphere. In the cloud-free atmosphere, the shortwave fluxes stemming from the sun can be absorbed and/or scattered by gaseous and solid constituents, such as water vapor and other radiatively active gases as well as aerosols. Radiatively active gases are also mainly responsible for the longwave emission of the cloud-free atmosphere towards space and the Earth’s surface. For the quantification of the effects of clouds on the Earth’s energy balance, the precise magnitudes of the radiative fluxes both under “all-sky” (including clouds) and “clear-sky” (excluding clouds) conditions need to be known (Ramanathan 1987).

Thanks to the sophisticated satellite observing systems in operation since the turn of the millennium, the radiative fluxes in and out of the climate system at the TOA are now well determined (Loeb et al. 2012, 2018). This allows also an accurate estimation of the impact of clouds on the TOA radiation budget through the comparison of satellite measurements representing all-sky and clear-sky conditions, and an adequate assessment of their representation in climate models (e.g., Potter and Cess 2004; Wang and Su 2013). The magnitudes of radiative fluxes at the Earth’s surface, however, both under all-sky and clear-sky conditions, are not known with the same accuracy, since they cannot be directly measured from satellites (Kiehl and Trenberth 1997; Hatzianastassiou et al. 2005; Wild et al. 2006, 2013; Trenberth et al. 2009; Trenberth and Fasullo 2012; Stephens et al. 2012; L’Ecuyer et al. 2015; Pfeifroth et al. 2018). Similarly, the radiation budgets simulated by the latest generation of global climate models (GCMs) participating in the Coupled Model Intercomparison Project Phase 5 (CMIP5) show large discrepancies already on a global mean basis particularly at the Earth’s surface, and this not only under all-sky, but also under clear-sky conditions, as will be shown in the present study.

In previous studies (Wild et al. 2013, 2015) we made an attempt to better constrain the radiative fluxes in the CMIP5 models under all-sky conditions, using to the extent possible the information contained in surface radiation measurement records. In the present, complementary study we specifically focus on the clear-sky radiative fluxes. Thereby, we will make use of clear-sky reference climatologies that we established from the high-quality radiation records measured at worldwide distributed stations from the Baseline Surface Radiation Network (BSRN, Ohmura et al. 1998; Driemel et al. 2018). We will then use these newly established references to assess the clear-sky fluxes simulated by the CMIP5 models.

**Fig. 1:**
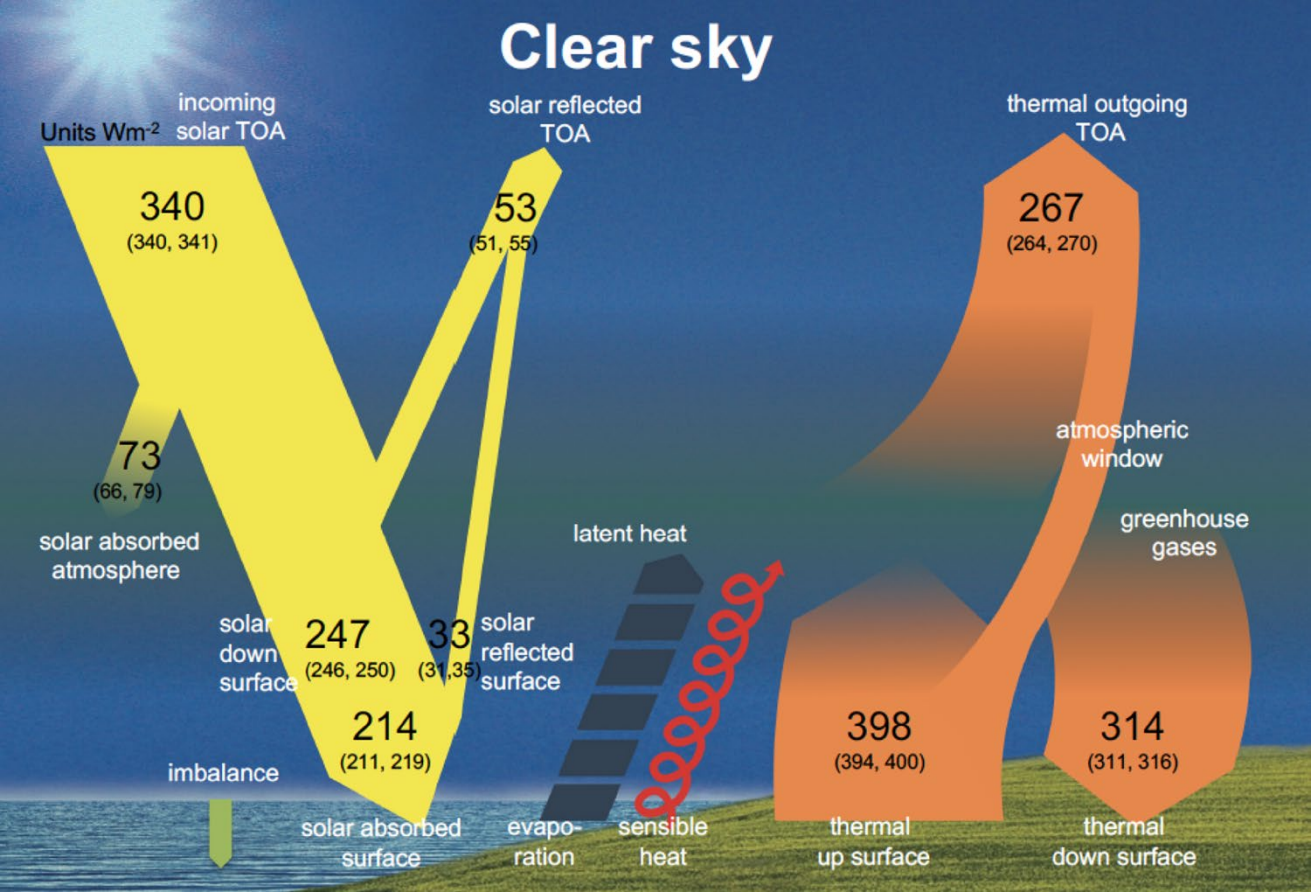
Schematic representation of the global annual mean energy balance of the Earth under cloud-free conditions. Numbers indicate best estimates for the magnitudes of the globally averaged clear-sky energy balance components together with their uncertainty ranges, representing present day climate conditions at the beginning of the new millennium. Estimates based on discussion in Sect. 6. Units Wm^−2^. Note that the cloud-free energy balance shown here is not the balance that Earth would achieve in equilibrium when no clouds could form. It rather represents the global mean fluxes as determined solely by removing the clouds but otherwise retaining the entire atmospheric structure (i.e. the GCM representation of clear-sky fluxes, also known as Method II according to Cess and Potter 1987). Thus, the TOA budget is not closed

We will further use the emerging bias structure of the CMIP5 clear-sky fluxes with respect to the BSRN records to infer best estimates for the global mean surface shortwave and longwave clear-sky fluxes. We will then incorporate these estimates, together with TOA flux estimates from satellites, to establish a diagram of the global energy balance under cloud-free conditions, as presented in Fig. 1. By combining these estimates with the corresponding all-sky estimates determined in our earlier studies we will finally quantify the global cloud radiative effects not only at the TOA, but also within the atmosphere and at the surface.

## Climate models

In the present study we assess the clear-sky radiation climatologies as simulated by a variety of GCMs from the Coupled Model Intercomparison Project Phase 5 (CMIP5), which provided the basis for the most recent (5th) IPCC assessment report (IPCC 2013). They correspond to the 43 models listed in Table 1 of Wild et al. (2015), except for the following models which could not, or only partly, be considered in the present study due to incomplete clear-sky datasets: model versions CMCC-CESM, CMCC-CM, CMCC-CMS of the Centro Euro-Mediterraneo per I Cambiamenti Climatici (no surface shortwave and longwave clear-sky fluxes), CNRM-CM5 from the Centre National de Recherches Meteorologiques (wrong partitioning of shortwave clear-sky fluxes into downward and upward components over land), FGOALS-g2 from the Institute of Atmospheric Physics (corrupted surface downward clear-sky fluxes at poles, no surface shortwave clear-sky upward fluxes), and two versions of the Community Earth System Model, CESM1-CAM5 and CESM1-CAM5.1-FV2 (no surface clear-sky downward longwave fluxes). This leaves 38 and 37 CMIP5 models for the analysis of the shortwave and longwave clear-sky fluxes, respectively.

**Table 1:** BSRN observation sites used in this study, alphabetically ordered according to their shortcuts, together with their associated country/region, latitudes (positive north/negative south), longitudes (positive east/negative west), altitudes (m) and their climatological annual means of clear-sky downward shortwave and longwave radiation (Wm^−2^), determined as described in Sect. 3

Station shortcut	Station name	Country/region	Latitude	Longitude	Altitude	Clear-sky SW down	Clear-sky LW down
ALE	Alert	Lincoln Sea, Canada	82.49	− 62.42	127	126.4	174.4
ASP	Alice Springs	Macdonnell Ranges, NT, Australia	− 23.798	133.888	547	291.5	
BAR	Barrow	Alaska, USA	71.323	− 156.607	8	128.1	
BER	Bermuda	Bermuda	32.267	− 64.667	8	260.5	347.1
BIL	Billings	Oklahoma, USA	36.605	− 97.516	317	251.9	310.1
BON	Bondville	Illinois, USA	40.0667	− 88.3667	213	241.9	
BOS	Boulder	Colorado, USA	40.125	− 105.237	1689	252.5	
BOU	Boulder	Colorado, USA	40.05	− 105.007	1577	253.5	264.4
BRB	Brasilia	Brasilia City, Distrito Federal, Brazil	− 15.601	− 47.713	1023	283.8	342.8
CAB	Cabauw	The Netherlands	51.9711	4.9267	0	195.3	287.2
CAM	Camborne	United Kingdom	50.2167	− 5.3167	88	202.6	
CAR	Carpentras	France	44.083	5.059	100	222.2	300.0
CLH	Chesapeake Light	North Atlantic Ocean, USA	36.905	− 75.713	37	249.5	309.4
CNR	Cener	Spain, Sarriguren, Navarra	42.816	− 1.601	471	231.0	290.0
COC	Cocos Island	Cocos (Keeling) Islands	− 12.193	96.835	6	299.4	
DAA	De Aar	South Africa	− 30.6667	23.993	1287	273.8	299.7
DAR	Darwin	Australia	− 12.425	130.891	30	292.5	394.9
DOM	Concordia Station, Dome C	Antarctica	− 75.1	123.383	3233	160.4	
DRA	Desert Rock	Nevada, USA	36.626	− 116.018	1007	263.5	
E13	Southern Great Plains	Oklahoma, USA	36.605	− 97.485	318	251.3	309.3
EUR	Eureka	Ellesmere Island, Canadian Arctic	79.989	− 85.9404	85	122.7	
FLO	Florianopolis	South Atlantic Ocean, Brazil	− 27.6047	− 48.5227	11	268.6	346.9
FPE	Fort Peck	Montana, USA	48.3167	− 105.1	634	214.3	
FUA	Fukuoka	Japan	33.5822	130.3764	3	253.4	320.8
GCR	Goodwin Creek	Mississippi, USA	34.2547	− 89.8729	98	252.4	
GVN	Georg von Neumayer	Dronning Maud Land, Antarctica	− 70.65	− 8.25	42	155.3	180.8
ILO	Ilorin	Nigeria	8.5333	4.5667	350	285.0	
ISH	Ishigakijima	Japan	24.3367	124.1644	6	270.4	373.9
KWA	Kwajalein	North Pacific Ocean	8.72	167.731	10	296.6	404.6
LAU	Lauder	New Zealand	− 45.045	169.689	350	232.2	
LER	Lerwick	Shetland Island, United Kingdom	60.1389	− 1.1847	80	167.1	
LIN	Lindenberg	Germany	52.21	14.122	125	193.9	272.5
MAN	Momote	Papua New Guinea	− 2.058	147.425	6	299.8	407.8
MNM	Minamitorishima	Minami-Torishima, Japan	24.2883	153.9833	7	277.6	378.5
NAU	Nauru Island	Nauru	− 0.521	166.9167	7	302.6	408.3
NYA	Ny-Ålesund	Ny-Ålesund, Spitsbergen	78.925	11.93	11	125.4	215.4
PAL	Palaiseau, SIRTA Observatory	France	48.713	2.208	156	206.7	285.4
PAY	Payerne	Switzerland	46.815	6.944	491	214.5	277.2
PSU	Rock Springs	Pennsylvania, USA	40.72	− 77.9333	376	241.6	
PTR	Petrolina	Brazil	− 9.068	− 40.319	387	289.5	361.4
REG	Regina	Canada	50.205	− 104.713	578	211.6	236.4
SAP	Sapporo	Japan	43.06	141.3286	17	228.5	277.2
SBO	Sede Boqer	Israel	30.8597	34.7794	500	265.2	305.9
SMS	São Martinho da Serra	Brazil	− 29.4428	− 53.8231	489	268.4	
SOV	Solar Village	Saudi Arabia	24.91	46.41	650	279.0	
SPO	South Pole	Antarctica	− 89.983	− 24.799	2800	141.2	87.6
SXF	Sioux Falls	South Dakota, USA	43.73	− 96.62	473	231.5	
SYO	Syowa	Cosmonaut Sea, Antarctica	− 69.005	39.589	18	159.1	182.4
TAM	Tamanrasset	Algeria	22.7903	5.5292	1385	297.3	
TAT	Tateno	Japan	36.0581	140.1258	25	252.7	306.8
TIK	Tiksi	Siberia, Russia	71.5862	128.9188	48	133.3	
TOR	Toravere	Estonia	58.254	26.462	70	173.1	
XIA	Xianghe	China	39.754	116.962	32	243.6	

As in our previous studies (Wild et al. 2013, 2015) the model-calculated flux climatologies stem from the “historical all forcings” experiments, which aim at reproducing the evolution of climate over the twentieth century as realistically as possible, considering all major natural and anthropogenic radiative forcings. These include changes in atmospheric greenhouse gases, aerosol loadings (tropospheric and stratospheric volcanic), solar output, and land use. Most of the CMIP5 historic experiments cover the period from the mid nineteenth century up to 2005.

The GCM clear-sky fluxes are determined at every time-step irrespective of the presence of clouds. During cloudy conditions, clear-sky fluxes are calculated by removing the clouds in the radiative transfer calculations, but otherwise retaining the atmospheric input structure (e.g. profiles of water vapor, temperature, aerosols) that prevail during these cloudy conditions. This is also known as “Method II” according to Cess and Potter (1987) and Potter et al. (1992), whereas their definition of “Method I” refers to clear-sky climatologies which are only composed of truly cloud-free episodes. Clear-sky flux climatologies were established from the CMIP5 models as 5-year averages over the final complete years of their “historical all forcings” experiments, which correspond to the years 2000–2004. Since the year to year variations in the model-calculated clear-sky fluxes are small (the median annual variation at the various BSRN sites, defined as difference between the 5 and 95 percentiles, amounts to 2 Wm^−2^), a 5-year mean gives a representative depiction of the model clear-sky flux climatologies at the beginning of the 21th century. To derive a correction, which ensures an adequate comparison between model-calculated (according to Method II above) and observed (according to Method I) shortwave clear-sky flux climatologies, we additionally made use of the multi-century unforced control simulations of the CMIP5 models, as well as an in-house simulation with the model MPI-ESM-LR (see Sect. 4). The latter allowed to store and investigate the clear-sky fluxes at higher temporal resolution (daily data) than provided by the CMIP5 archive (only monthly data).

A detailed description of the models participating in CMIP5 is provided on the CMIP5 web-pages of the Program for Climate Model Diagnosis and Intercomparison (PCMDI) (http://www-pcmdi.llnl.gov/). From most CMIP5 models multiple simulations of the historic experiments are available, which only differ in their initial conditions (ensemble experiments). However, the different ensemble members from a specific model hardly differ in terms of their clear-sky radiative flux climatologies (by less than 1 Wm^−2^ on average), and the results presented in this study were found not to be sensitive to the choice of a particular ensemble member from a specific model. Therefore, results from only one ensemble realization of each model are presented in this study. This also avoids giving too much weight to specific models with large numbers of ensembles.

## Clear-sky reference climatologies

The observational reference data for the clear-sky fluxes at the Earth’s surface are derived from the records of the Baseline Surface Radiation Network (BSRN, Ohmura et al. 1998; Driemel et al. 2018). BSRN is a worldwide network of radiation sites measuring at highest possible accuracy with well-calibrated instruments and known accuracy, which can serve as anchor sites for a variety of applications. BSRN became operational in the early 1990s with a few sites and has gradually been growing to contain now more than 60 sites in different climate zones, which report their data to the BSRN Archive at the Alfred Wegener Institute (AWI) (http://www.bsrn.awi.de/). The BSRN data are recorded and stored at high temporal resolution (minute data), which is a prerequisite for the application of clear-sky detection algorithms as outlined below. BSRN sites are further requested to record shortwave radiation not only in terms of its total flux (measured with a pyranometer), but also separately in terms of the direct shortwave flux (measured with a pyrheliometer) and the diffuse shortwave flux (measured with a shaded pyranometer).

The availability of both direct and diffuse measurements is indispensable for the detection of cloud-free conditions, as clouds substantially modify the ratio between these two components. The Long and Ackerman (2000) clear-sky detection algorithm for shortwave radiation that we apply in this study makes use of this behaviour, as well as of the high temporal resolution of the data. Specifically, this algorithm analyses the magnitude and variability of the minute time-series of total (global) and diffuse shortwave irradiance periods to infer clear (i.e. cloudless) sky episodes. The identified clear-sky data at minute resolution are then used on a daily basis to empirically fit functions using the cosine of the solar zenith angle as the independent variable for days that exhibit the minimum amount of clear-sky times across the necessary range of solar zenith angles as defined by Long and Ackerman (2000). The fit coefficients are interpolated through time for days that cannot be adequately fitted, and then the coefficients (fitted or interpolated) are used to calculate continuous estimates of the clear-sky downward total, diffuse and direct shortwave radiation. The methodology requires some modification for climates such as the tropical western Pacific characterized by persistent cloudiness as detailed in Long and Gaustad (2004). As shown in Long and Ackerman (2000), the uncertainty of the clear-sky estimates due to interpolation is about of the same magnitude as the total shortwave measurements themselves for the instantaneous values. The long-term averaging of these values as used in the present study significantly decreases the uncertainties. Following the procedure as described above, clear-sky climatologies of surface downward shortwave radiation were produced at 53 BSRN sites which provide multiyear shortwave records that allow to establish climatologies (Table 1) (Hakuba et al. 2017). The periods covered by these reference climatologies do not necessarily exactly match the ones of the model climatologies (which is not a strict requirement due to the non-deterministic nature of the modelling setups), yet both observational and model climatologies can be considered as representative of the beginning of the 21th century.

The clear-sky detection algorithm for the longwave radiation is based on Long and Turner (2008), and takes into account the temporal variability of downward longwave radiation, as well as the difference between the measured ambient air temperature and effective sky brightness temperature calculated from the longwave measurements. The detected “effective” clear-sky data are used along with the previously detected shortwave clear-sky data to fit functions using collocated air temperature and humidity measurements as the independent variable in the formulation developed by Brutsaert (1975). These longwave detected periods are labelled “effective” clear-sky because the broadband downwelling longwave is insensitive to high, cold, thin clouds, thus the detected clear-sky times represent primarily periods with no low- and mid-level clouds. Similar to the shortwave clear-sky method, the fit coefficients are interpolated through time and used to produce continuous estimations of clear-sky longwave fluxes. Following this procedure, the data availability allowed the construction of clear-sky climatologies of surface downward longwave radiation at 31 BSRN sites (Table 1).

The TOA clear-sky references referred to in this study are obtained from the satellite-based Energy Balanced and Filled (EBAF) dataset from the Clouds and the Earth’s Radiant Energy System (CERES, Wielicki et al. 1996) program, CERES-EBAF Edition 4.0 (Loeb et al. 2018).

## Shortwave clear-sky fluxes

### Global budgets

**Fig. 2:**
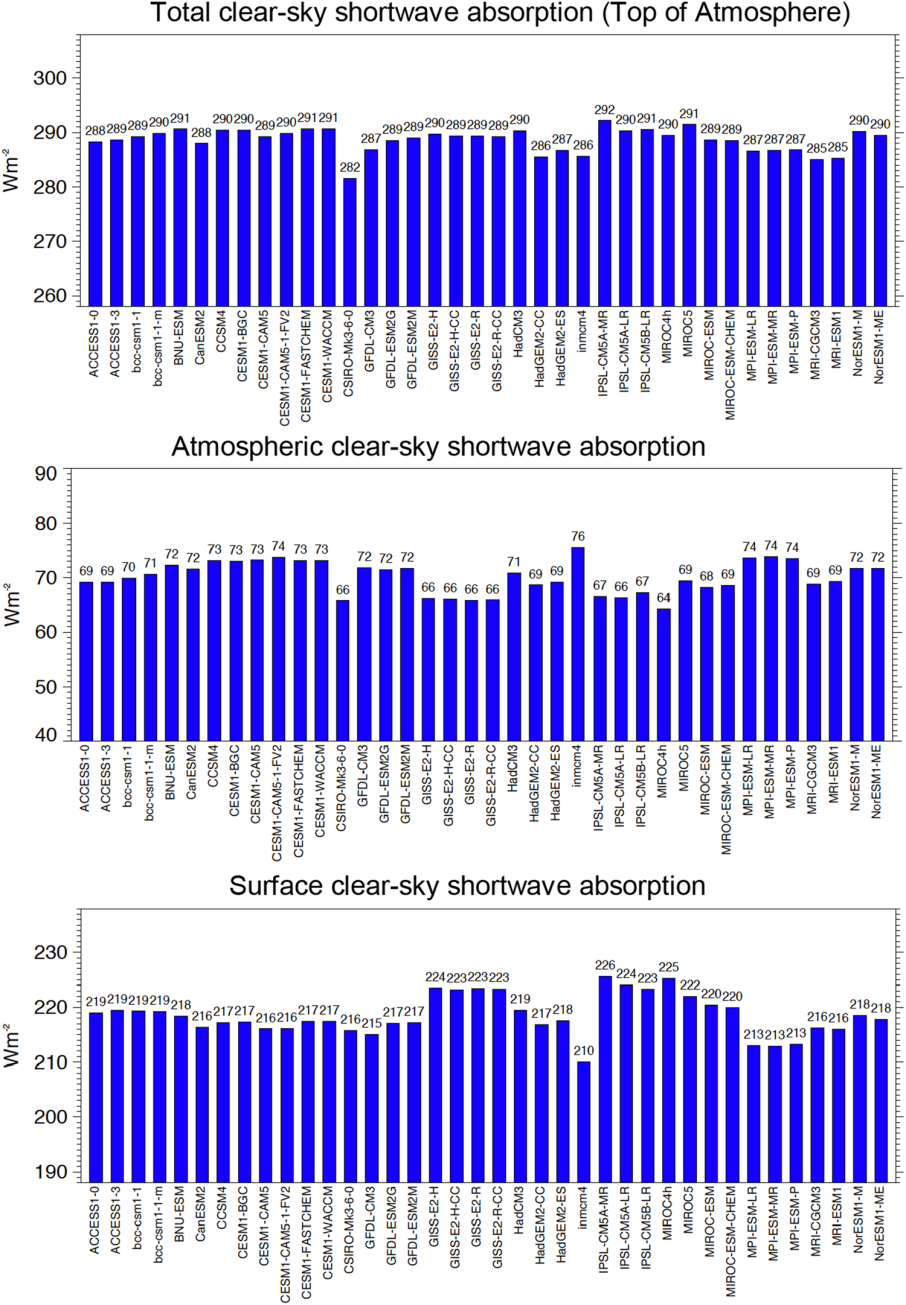
Global annual mean clear-sky shortwave radiation budgets of the total climate system (Top of Atmosphere), of the atmosphere (atmospheric absorption) and at the surface (surface absorption) in 38 GCMs participating in the Coupled Model Intercomparison Project Phase 5 (CMIP5). Averages taken over the period 2000–2004 of the “historical all forcings” experiments. Clear-sky fluxes in climate models are determined using Method II according to Cess and Potter (1987). Units Wm^−2^

Figure 2 shows the global annual mean clear-sky budgets as simulated by 38 CMIP5 GCMs at the surface (bottom panel), within the atmosphere (middle panel) and at the TOA (upper panel). The budgets at the TOA that govern the total amount of clear-sky absorption in the climate system, are to some extent tuned to match the CERES reference value, given at 287 Wm^−2^ for the global mean TOA shortwave clear-sky absorption. Accordingly, the corresponding quantity in the CMIP5 multi-model mean, at 288.6 Wm^−2^, closely matches the CERES reference (Table 2). Between the individual models, this quantity varies in a range of 10 Wm^−2^, with a standard deviation of 2.1 Wm^−2^, and with a maximum deviation of 5 Wm^−2^ from the CERES reference value (Table 2; Fig. 2 upper panel).

**Table 2:** Global annual mean estimates of the magnitude of various energy balance components under clear-sky and all-sky conditions at the TOA, within the atmosphere and at the surface

Energy balance component	Reference estimates	CMIP5 multi-model mean	CMIP5 model range	CMIP5 model SD
**TOA**				
Solar down TOA	340	341.3	3.4	0.8
Solar up all-sky TOA	− 100	− 102.0	12.6	3.1
Solar net all-sky TOA	240	239.2	11.2	3.0
Solar up clear-sky TOA	− 53	− 52.6	11.2	2.3
Solar net clear-sky TOA	287	288.6	10.6	2.1
Solar CRE TOA	− 47	− 49.3	14.0	3.5
Thermal up all-sky TOA	− 239	− 238.0	11.7	2.9
Thermal up clear-sky TOA	− 267	− 263.3	12.9	3.3
Thermal CRE TOA	28	24.9	12.6	3.5
Net CRE TOA	− 19	− 24.1	15.5	3.9
**Atmosphere**				
Solar net all-sky atmosphere	80	74.7	9.9	2.8
Solar net clear-sky atmosphere	73	70.1	11.3	2.9
Solar CRE atmosphere	7	4.3	8.8	1.6
Thermal net all-sky atmosphere	− 183	− 179.8	22.5	3.8
Thermal net clear-sky atmosphere	− 183	− 179.1	15.0	2.9
Thermal CRE atmosphere	0	− 0.5	19.5	3.5
Net CRE atmosphere	7	4.7	18.9	4.1
**Surface**				
Solar down all-sky surface	185	189.6	15.8	4.7
Solar up all-sky surface	− 25	− 24.6	10.5	2.3
Solar net all-sky surface	160	165.0	12.2	3.8
Solar down clear-sky surface	247	249.7	13.3	3.6
Solar up clear-sky surface	33	31.1	12.8	2.9
Solar net clear-sky surface	214	218.5	15.5	3.6
Solar CRE surface	− 54	− 53.5	16.7	4.1
Thermal down all-sky surface	342	340.1	18.5	4.3
Thermal up all-sky/clear-sky surface	− 398	− 398.7	10.7	2.6
Thermal net all-sky surface	− 56	− 58.6	15.7	3.2
Thermal down clear-sky surface	314	314.7	25.8	5.5
Thermal net clear-sky surface	− 84	− 83.9	15.9	3.7
Thermal CRE surface	28	25.3	13.3	3.3
Net CRE surface	− 26	− 28.6	24.4	4.8

Given are the reference estimates, together with the CMIP5 model estimates in terms of their multi-model means, their ranges and their standard deviations (SD). Clear-sky reference estimates and Cloud Radiative Effects (CRE) derived in the present study, all-sky reference estimates from Wild et al. (2015). TOA fluxes derived from CERES-EBAF (Loeb et al. 2018). Units Wm^−2^. Clear-sky model and reference estimates determined following Method II according to Cess and Potter (1987)

No similarly trusted reference values are available for the global mean shortwave absorption under cloud-free conditions at the Earth surface. Accordingly, model estimates differ in a larger range (16 Wm^−2^) and with a larger standard deviation (3.6 Wm^−2^) than their TOA counterparts, despite the absolute values being lower by about 25% (Table 2; Fig. 2 lower panel). The global mean absorption of shortwave radiation in the cloud-free atmosphere also varies between different models in a range of 11 Wm^−2^ (Fig. 2, middle panel), which corresponds to 17% of their absolute values spread around 70 Wm^−2^, and with a standard deviation of 2.9 Wm^−2^ (Table 2).

**Fig. 3:**
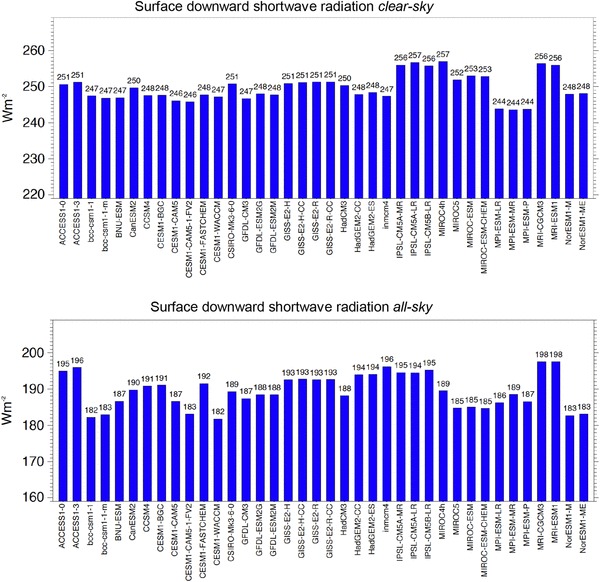
Global annual mean downward shortwave radiation at Earth’s surface under present day climate calculated by 38 CMIP5 models under clear-sky conditions (upper panel) and under all-sky conditions (lower panel). Averages taken over the period 2000–2004 of the “historical all forcings” experiments. Clear-sky fluxes are determined using Method II according to Cess and Potter (1987). Units Wm^−2^

The large spread amongst the models remains if we consider the downward rather than the absorbed clear-sky fluxes at the surface which can directly be compared to the upward-oriented radiation sensors of the surface stations. In their global means, these clear-sky fluxes vary in a range of 13 Wm^−2^, with a standard deviation of 3.7 Wm^−2^ (Fig. 3 upper panel, Table 2). Interestingly, this inter-model spread is not that much smaller than the all-sky equivalent of the downward shortwave radiation, despite neglecting cloud effects (Fig. 3 lower panel, Table 2).

### Surface flux assessment

To better constrain the unsatisfactory large spread in the model calculated shortwave clear-sky fluxes at the surface noted above, we use the clear-sky reference climatologies established from records compiled at 53 BSRN sites (see Sect. 3, Table 1) and perform a pointwise comparison with the corresponding flux fields from the CMIP5 models. Thereby the model-calculated fields of clear-sky downward shortwave radiation were interpolated to the coordinates of the BSRN stations taking into account the four distance-weighed surrounding grid points. The random error induced by the comparison of point observations with gridded shortwave flux products with a resolution of 1° as typically found in GCMs has been estimated by Hakuba et al. (2013, 2014) to be on the order of 3 Wm^−2^ on a climatological timescale, which is attributed to the subgrid-scale variability not resolved by the GCM grid. This applies for all-sky fluxes and should be rather an upper limit for clear-sky fluxes due to their spatially smoother fields.

The comparison is complicated by the fact that the monthly shortwave clear-sky BSRN reference climatologies are derived from measurements under truly cloud-free conditions, whereas the GCM clear-sky fluxes are calculated continuously at every time-step solely by removing the clouds, yet otherwise retaining the prevailing atmospheric composition (e.g. water vapor, temperature, aerosols) during the cloudy conditions. As mentioned in Sect. 2, the former method of determining the clear-sky fluxes is also known in the literature as Method I, whereas the latter is referred to as Method II (Cess and Potter 1987; Potter et al. 1992). These different definitions of clear-skies in models and observations give rise to potentially spurious biases in the GCMs with respect to the reference climatologies due to the additional sampling of clear-sky fluxes calculated under atmospheric conditions representative for cloudy situations. Thereby, the different sampling may result in a wet bias in the GCMs compared to the clear-sky reference climatologies, which may induce some spurious additional shortwave atmospheric absorption, thus potentially leading to downward clear-sky shortwave fluxes that are biased artificially somewhat low compared to “true” clear-sky conditions. To obtain an estimate of the magnitude of this potential spurious sampling bias, an obvious procedure would be to compose clear-sky climatologies from the model fluxes only out of situations when true clear-sky conditions prevail. However, this would require access to raw model output at high temporal resolution (ideally at the model time step), yet this is not available from the CMIP5 archive. To nevertheless get a sense of whether the different definitions of clear-skies in models and observations could be of any relevance, we applied the following methodology (Ott 2017): We took from all CMIP5 models used in the present study monthly data from their very long unforced control runs, which typically cover many centuries of integration. Within these multi-century simulations of each model, we searched for the specific month with the least cloud amount, for each month of the year separately (i.e. the January with the least cloud amount out of all Januarys in the control run (typically several hundreds), and similar for each other month of the year through to December). The aim thereby was to approximate as best as we can on a monthly basis true clear-sky conditions. We then took the associated model clear-sky fluxes of these particularly cloud-free months, and compared them to the corresponding long term climatological mean clear-sky fluxes as averaged over the entire control run. This can give an idea to what extent the model-calculated monthly fluxes under as cloudless as possible conditions deviate from the corresponding clear-sky fluxes calculated under all types of cloud conditions. These deviations have been determined for each model at each BSRN station, and were subsequently used as corrections to adjust the simulated downward shortwave flux climatologies individually for each model and each station. On average, the deviations were found to be relatively modest, on the order of 2 Wm^−2^, with the clear-sky fluxes of the months with lowest cloudiness systematically slightly higher than under all type of cloud conditions, as expected due to the dryer atmosphere of the former.

We had also the chance to double check the magnitude of this adjustment further, by analysing daily rather than monthly data from a 150-year long control simulation that we performed in-house with one particular model, the MPI-ESM-LR. Thereby we compared model-calculated clear-sky fluxes from the most cloudless days with the respective clear-sky fluxes averaged over all corresponding days from this simulation, for each day of the year. A similar deviation on the order of 2 Wm^−2^ was obtained on average over all BSRN sites. This increases our confidence in the magnitude of this spurious sampling bias, and in the associated adjustments we applied.

**Fig. 4:**
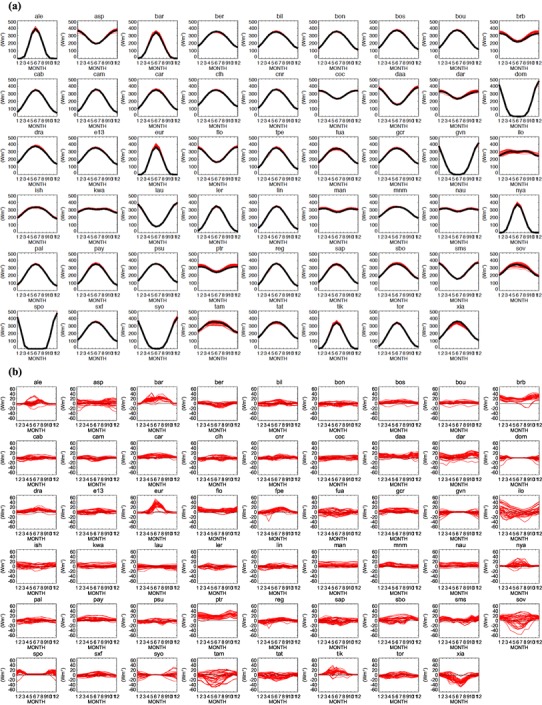
**a** Annual cycles of clear-sky downward surface shortwave radiation climatologies measured at 53 BSRN sites (black lines) and simulated by 38 CMIP5 climate models (red lines). **b** Differences between the individual model-calculated and observed annual cycles. Model and reference climatologies representative for the beginning of the 21th century. Station abbreviations explained in Table 1. Units Wm^−2^

Model-calculated climatologies of the annual cycles of downward surface shortwave clear-sky radiation from the 38 CMIP5 models, slightly adjusted according to the above procedure to ensure a proper comparison with the reference climatologies, are compared in Fig. 4 with the BSRN reference climatologies at 53 stations. In Fig. 4a the mean annual cycles of the 38 models are shown in red, whereas the annual cycles of the BSRN reference climatologies are given in black. Figure 4b displays the associated differences between the simulated and observed mean annual cycles for each model. Note the smooth shapes of the annual cycles in Fig. 4a which are characteristic for clear-sky climatologies. Overall the models reproduce the reference annual cycles reasonably well, with monthly biases of no more than 10–20 Wm^−2^. Only at a few stations, large differences can be noted, mostly in the summer months with high absolute values. Substantial model spread can be seen at desert sites which are heavily influenced by aerosols [ilo: Ilorin (Nigeria), tam: Tamanrasset (Algeria), sov: Solar Village (Saudi Arabia)].

**Fig. 5:**
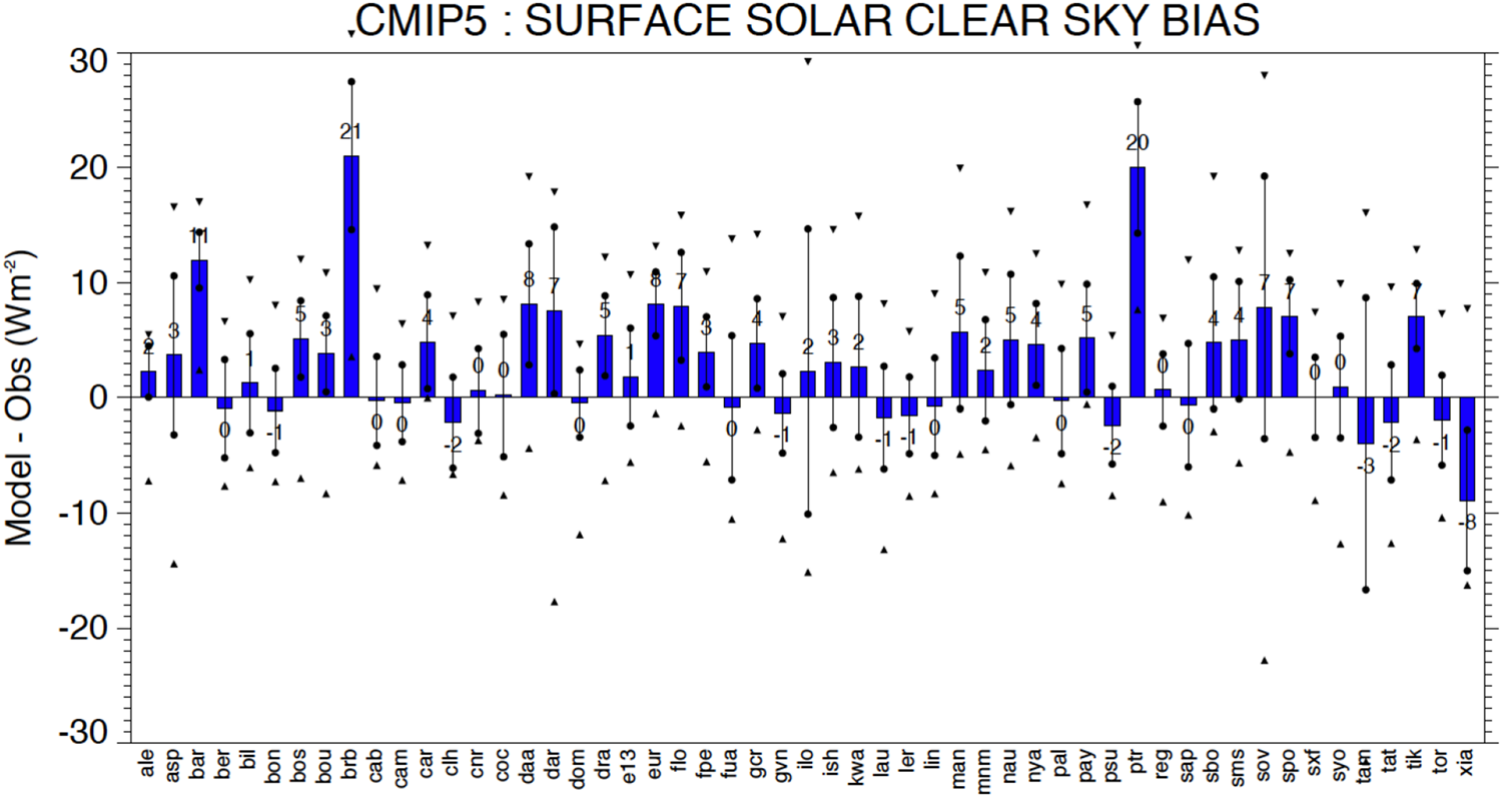
Multi-model mean biases (model-observations) in annual mean downward surface clear-sky shortwave radiation at 53 different BSRN sites (blue bars). The spread of individual model biases is further indicated with a vertical line covering ± one standard deviation, and with the maximum positive and negative model biases given as triangle symbols. Station abbreviations explained in Table 1. Units Wm^−2^

The climatological annual mean differences between model-calculated and observed downward surface shortwave clear-sky radiation at each of the 53 BSRN sites are shown in Fig. 5. The blue bars denote multi-model mean differences between the model-calculated and reference clear-sky climatologies at each individual BSRN station. The spread of model-calculated values at each site is further indicated by vertical lines covering ± one standard deviation, and by the maximum positive and negative model biases given as triangle symbols. There is an overall tendency that the models overestimate the downward surface shortwave clear-sky fluxes compared to the BSRN references, with a multi-model mean overestimation at 35 out of the 53 sites (two-third of the sites). The multi-model mean and median overestimation with respect to the 53 sites is 2.9 and 2.3 Wm^−2^, respectively. There is, however, a considerable fraction, namely 20 sites with multi-model mean biases of no more than 1 Wm^−2^. This is remarkable given the complex processing that stands behind both model and reference data. At two sites, both located in Brazil (brb and ptr), the multi-model mean differences are particularly high. Although there is the possibility that the models underestimate the absorption by aerosols from biomass burning in these areas we cannot entirely rule out the possibility of a measurement problem at these remote sites. Without considering these two sites, the abovementioned multi-model mean bias reduces by 0.7 Wm^−2^ to 2.3 Wm^−2^, whereas the median bias is hardly affected.

**Fig. 6:**
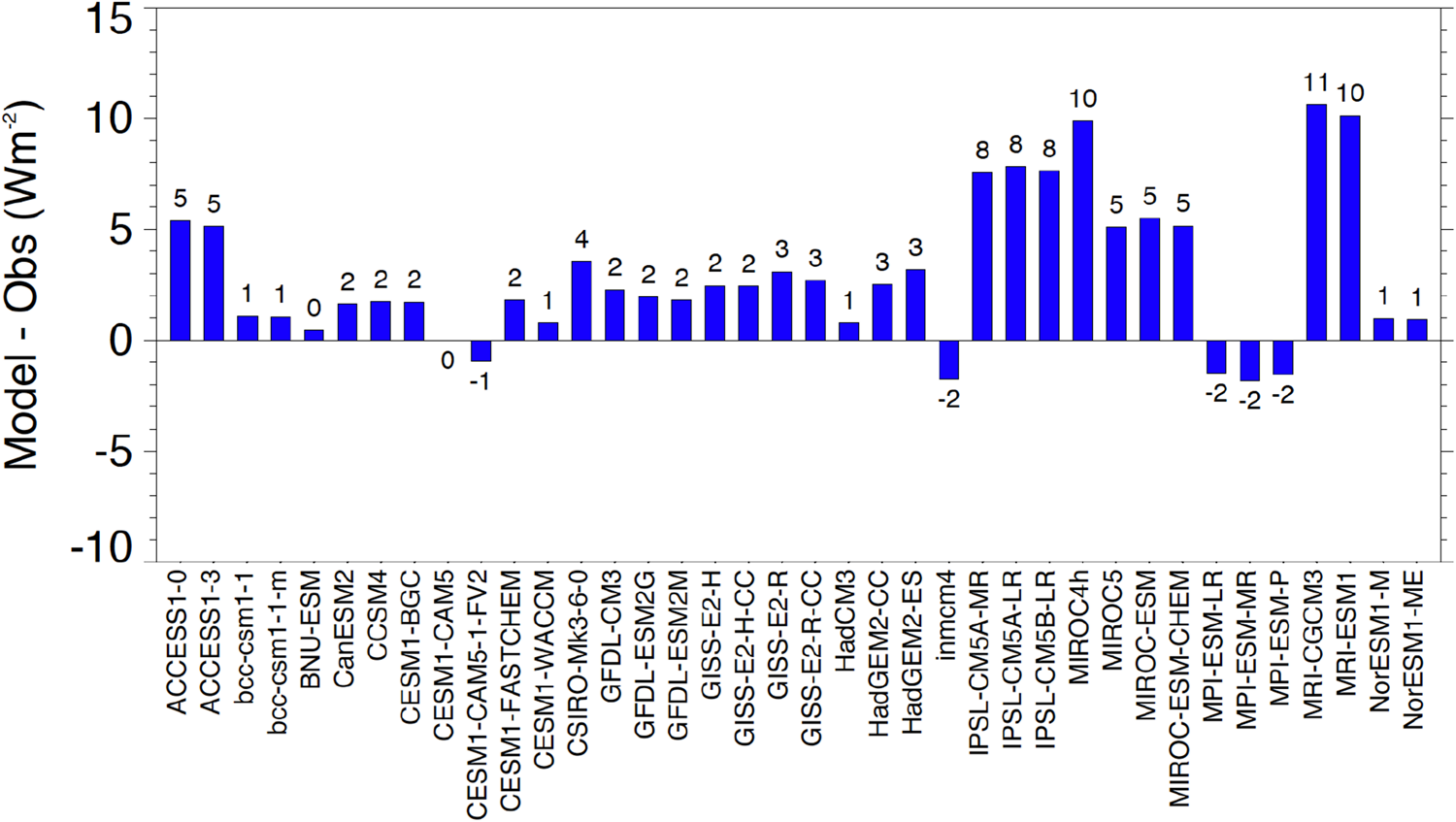
Biases (model-observations) in climatological annual mean downward clear-sky shortwave radiation at Earth’s surface calculated in 38 CMIP5 models averaged over 53 BSRN sites. Units Wm^−2^

The overall biases of the individual CMIP5 models when averaged over all 53 sites are given in Fig. 6. They largely vary between models, in a range of 13 Wm^−2^, which is not unexpected given the large model discrepancies already in their global mean downward surface shortwave clear-sky radiation (c.f. Fig. 3 upper panel). Most models (32 out of 38) show an overestimation. Averaged over all 38 models, this overestimation amounts to 2.9 Wm^−2^, while the median model overestimation is 2.2 Wm^−2^. Again, excluding the two Brazilian sites would reduce the overestimation by about 0.7 Wm^−2^. It is also noteworthy that more than half of the models show an overall bias compared to the BSRN references of no more than 2 Wm^−2^. The overestimation of the downward surface shortwave clear-sky radiation has been a long-standing issue in climate models, as inferred in earlier studies from fewer observational references and older models compared to the present study (Wild et al. 1995, 1998, 2006). An underestimation of water–vapor absorption in the GCM radiation codes, related to uncertainties in the spectroscopic absorption coefficients and the formulation of the near-infrared water vapor continuum, as well as inaccuracies in the representation of aerosol characteristics and burdens have been put forward as potential causes for the overestimation of the downward surface shortwave clear-sky radiation (Wild et al. 1998; Morcrette 2002; Pincus et al. 2015; Paynter and Ramaswamy 2012, 2014; Radel et al. 2015). This is also in line with findings of the Continual Intercomparison of Radiation Codes (CIRC, Oreopoulos and Mlawer 2010) and preceding radiation code intercomparison projects (Fouquart et al. 1991). While these intercomparison projects were useful to identify radiation codes which do not adequately represent well-established radiation physics [e.g., some radiation codes entirely neglect the N_2_O and CH_4_ absorption (Collins et al. 2006)], they may not be able to nail down uncertainties induced by incompletely understood physics such as aspects of the water vapor continuum or aerosol optical properties. Additional biases in the model-calculated downward surface shortwave clear-sky radiation could potentially be introduced by inadequacies in the simulated amount of water vapor (precipitable water) in the CMIP5 model atmospheres, which is used as input to the radiation schemes. Indeed there is recent evidence that the CMIP5 atmosphere are globally too dry, on average by about 6% when compared to satellite-derived references (Takahashi 2018). However, the impact of a dry bias of this magnitude on the downward surface shortwave clear-sky radiation is likely less than 1 Wm^−2^ (Wild 2009).

### Best estimate for the global mean downward surface shortwave clear-sky radiation

**Fig. 7:**
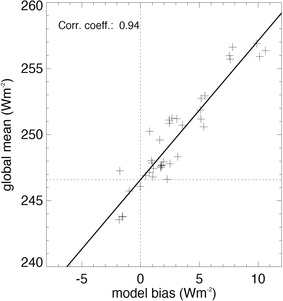
Global annual mean surface downward shortwave clear-sky radiation of 38 CMIP5 models (given as crosses) on the vertical axis versus their respective overall biases compared to 53 BSRN references on the horizontal axis. A “best estimate” for the surface downward shortwave clear-sky radiation is inferred at the intersect between the linear regression line and the zero bias line. Units Wm^−2^

We now make use of the bias structure of the CMIP5 models with respect to the BSRN references to infer a best estimate for the global mean downward surface shortwave clear-sky radiation. We thereby apply the methodology presented in Wild et al. (2013, 2015) for all-sky fluxes now to the clear-sky fluxes, and relate the diverging values in global mean downward surface shortwave clear-sky radiation of the various CMIP5 models (as given in Fig. 3) to their respective biases (as given in Fig. 6). Therefore, in Fig. 7, each cross represents one CMIP5 model with its global annual mean downward surface shortwave clear-sky radiation on the vertical axis and its overall climatological bias in this quantity compared to the 53 BSRN sites on the horizontal axis. A very tight connection between the global means and the corresponding biases of the downward surface shortwave clear-sky radiation calculated by the various models is evident, as reflected in a high correlation coefficient of 0.94. Thus, generally, the more a climate model overestimates the downward surface shortwave clear-sky radiation compared to the BSRN references, the higher is also its corresponding global mean value. A linear regression can then be used to determine the hypothetical global mean value that would correspond to a zero bias against the observational references (dashed lines in Fig. 7). The value that fits to the zero bias according to the linear regression is 246.6 (± 0.6) Wm^−2^ (2σ uncertainty given in the parentheses). We thus take this value as our best estimate for the global annual mean downward surface shortwave clear-sky radiation. Note that in the bias calculations the two suspicious Brazilian sites are included. Excluding these two sites puts the best estimate to 247.3 (± 0.6) Wm^−2^. This suggests a value near 247 Wm^−2^ as best estimate for the global annual mean downward surface shortwave clear-sky radiation. We further tested two different ways of calculating the regression in Fig. 7, namely a y-least squares and an orthogonal regression, but this had only a marginal influence of less than 0.2 Wm^−2^ on the best estimate derived here.

Unlike in the all-sky estimations in Wild et al. (2013, 2015), we cannot use the independent reference dataset from the Global Energy Balance Archive (GEBA, Wild et al. 2017) to doublecheck the robustness of our method with respect to the station distribution, as the observational records in GEBA are only available at monthly resolution and thus do not allow for the establishment of clear-sky climatologies. In Wild et al. (2013, 2015) we could show that for all-sky conditions our regression method to obtain the best estimates of the global mean downward fluxes is fairly robust with respect to the choice of independent reference datasets with differing station distributions. Robust results are expected from our regression method if no major common systematic geographical dependencies are present in the bias structures of the simulated flux climatologies, which could be missed by the BSRN network. We did not find any such dependencies in the clear-sky biases amongst the various CMIP5 models, such as latitudinal dependencies, differences in biases in polar versus tropical sites, or sites in maritime versus terrestrial environments etc., with the one exception of the outlier biases at the two Brazilian sites mentioned above. Therefore we believe that also our clear-sky estimates should be fairly robust, even though we cannot rule out the possibility that undetected outstanding biases in undersampled regions could affect the results.

As in our previous study (Wild et al. 2015), to obtain a conservative uncertainty range for our best estimate, we expand the 2σ regression uncertainty range given above to additionally cover all global mean clear-sky estimates from those GCMs which show only marginal biases compared to the surface stations. This accounts for the fact that any global mean estimate stemming from a GCM with only marginal overall biases against the surface sites strictly cannot be disqualified by our approach. Specifically, in Fig. 7 we note also global mean clear-sky downward solar radiation values spreading from 246 to 250 Wm^−2^ which belong to GCMs with marginal overall biases (defined here as smaller than 1 Wm^−2^), and which accordingly defines the uncertainty range of this quantity in Fig. 1.

## Longwave clear-sky fluxes

### Global budgets

**Fig. 8:**
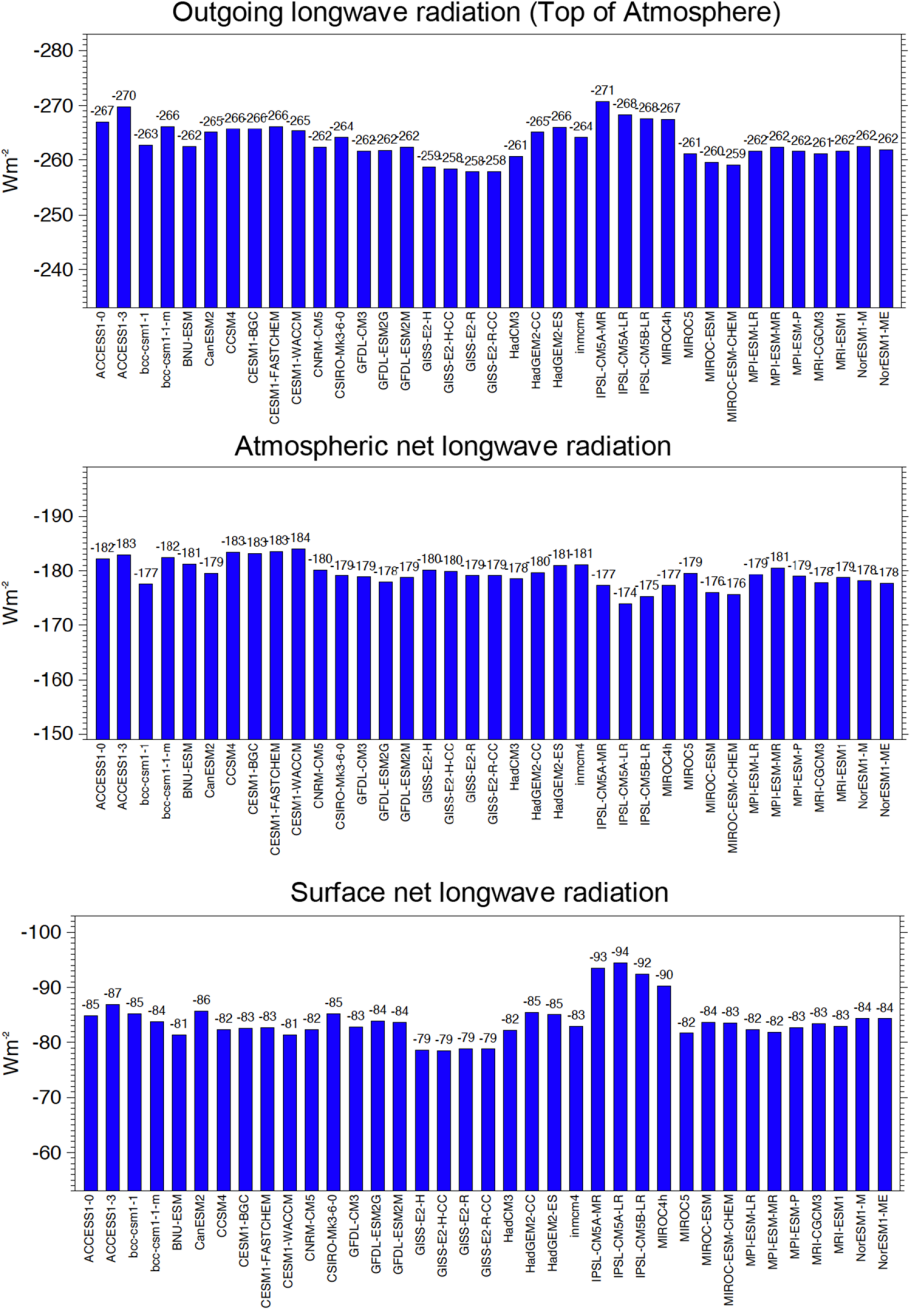
Global annual mean longwave radiation budgets for clear-sky conditions calculated by 37 CMIP5 models for present day climate (baseline 2000–2004). Net longwave radiation at the surface (lower panel), within the atmosphere (middle panel), and emitted to space (uppermost panel). Clear-sky fluxes in climate models are determined using Method II according to Cess and Potter (1987). Units Wm^−2^

Figure 8 displays the global annual mean net longwave budgets of 37 CMIP5 models for cloud-free conditions at the TOA (outgoing longwave radiation, OLR, upper panel), within the atmosphere (middle panel) and at the Earth’s surface (lower panel).

The multi-model global annual mean of the clear-sky OLR is 263 Wm^−2^, which is close to the estimate based on earlier CERES product releases (Ed 2 SYN1deg-Month) of 264 Wm^−2^ (Kato et al. 2013), which might have been used as tuning target during model developments, yet somewhat lower than more recent CERES reference estimates of 266 and 268 Wm^−2^ based on CERES Editions 2.6r and 4.0, respectively. The global mean clear-sky OLR varies between the 37 CMIP5 models in a range of 13 Wm^−2^ (Fig. 8 upper panel, Table 2). Again, this range is larger in the clear-sky longwave budget at the Earth’s surface (16 Wm^−2^, Fig. 8 lower panel, Table 2), where no generally accepted global reference estimates exist. Finally, the atmospheric clear-sky longwave budget of the 37 models is displayed in Fig. 8 (middle panel).

**Fig. 9:**
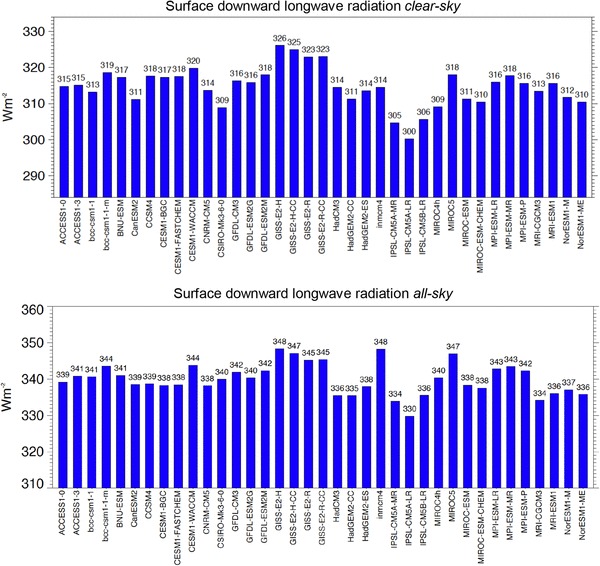
Global annual mean downward longwave radiation at Earth’s surface under present day climate calculated by 37 CMIP5 models under clear-sky conditions (upper panel) and under all-sky conditions (lower panel). Clear-sky fluxes are determined using Method II according to Cess and Potter (1987). Units Wm^−2^

The clear-sky component in the longwave that can be directly compared with surface observations is the surface downward longwave clear-sky radiation. Global annual means of this quantity for each CMIP5 model are shown in Fig. 9 (upper panel). Compared to the net longwave clear-sky radiation shown in Fig. 8 (lower panel) the discrepancies amongst the model-calculated global means is further enhanced, now covering a dissatisfactory large range of 26 Wm^−2^, with a standard deviation of 5.5 Wm^−2^ (Fig. 9 upper panel, Table 2). This spread is even larger than in the corresponding all-sky global means of the same component, where the model spread covers a smaller range and standard deviation of 19 and 4.3 Wm^−2^, respectively (Fig. 9 lower panel, Table 2). This suggests that cloud effects may help to mask some of the discrepancies found in the longwave clear-sky fluxes simulated by the various CMIP5 models. This issue has already been noted in earlier generations of climate models (AMIPII, CMIP3) (Wild 2008), and suggests that the longwave emission from the cloud-free atmosphere towards the Earth surface is a major cause for the inter-model differences seen in the (all-sky) downward longwave radiation.

### Surface flux assessment

In the following, the surface downward longwave clear-sky radiation fields of the CMIP5 GCMs are compared to the BSRN-derived clear-sky climatologies (c.f. Sect. 3). As with the shortwave, also the longwave clear-sky flux fields in the climate models stem from clear-sky calculations at each timestep, solely by removing the clouds if present, but otherwise retaining the atmospheric temperature and humidity structure (Method II according to Cess and Potter 1987), thus not only taking into account the atmospheric structure under true cloud-free conditions. However, unlike for the shortwave clear-sky assessment in Sect. 4.2, this states no problem for the comparison with the longwave BSRN reference climatologies. This is due to the fact that the algorithm that derives longwave clear-sky fluxes from the BSRN sites uses continuous measurements of air temperature and humidity regardless of whether clouds are present or not (Long and Turner 2008). Thus, the atmospheric conditions that enter the reference estimates are directly comparable to the ones that enter the GCM radiation codes, as both align with Method II, and thus there is no sampling issue. Therefore, unlike in the shortwave, no systematic sampling biases are apparent for the longwave clear-sky fluxes, and no further adjustments are required for an appropriate comparison of the simulated fluxes with the BSRN references.

**Fig. 10:**
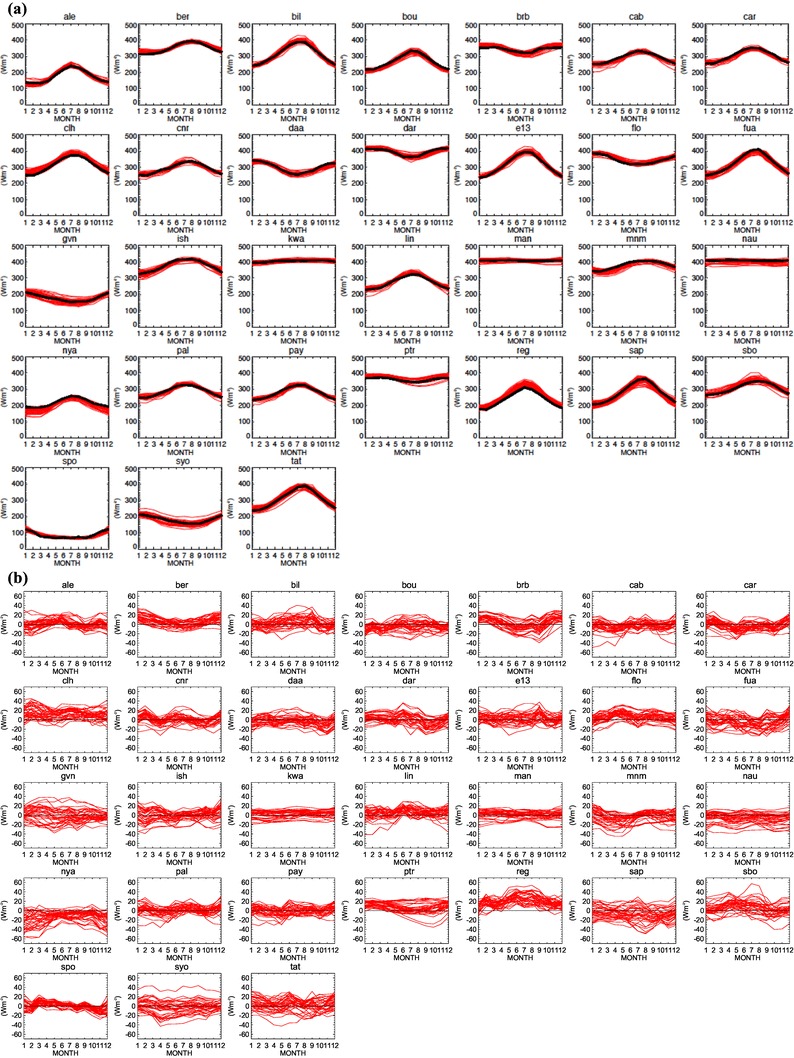
**a** Annual cycles of downward surface clear-sky longwave radiation climatologies measured at 31 BSRN sites (black lines) and simulated by 37 CMIP5 climate models (red lines). **b** Differences between the individual model-calculated and observed annual cycles. Model and reference climatologies representative for the beginning of the 21th century. Station abbreviations explained in Table 1. Units Wm^−2^

The simulated climatological mean annual cycles of downward surface longwave clear-sky radiation from the 37 CMIP5 models are compared in Fig. 10 with the BSRN reference climatologies at the 31 stations where such reference climatologies could be established (see Sect. 3, Table 1). In Fig. 10a, the annual cycles of the models are again shown in red, whereas the BSRN reference climatologies are given in black. The associated differences between the model-calculated and observed annual cycles are displayed in Fig. 10b. As in the shortwave (Fig. 4), the models overall reproduce the BSRN annual cycles reasonably well. This gives confidence in both model-simulated and observation-derived fluxes, which are completely independently obtained.

**Fig. 11:**
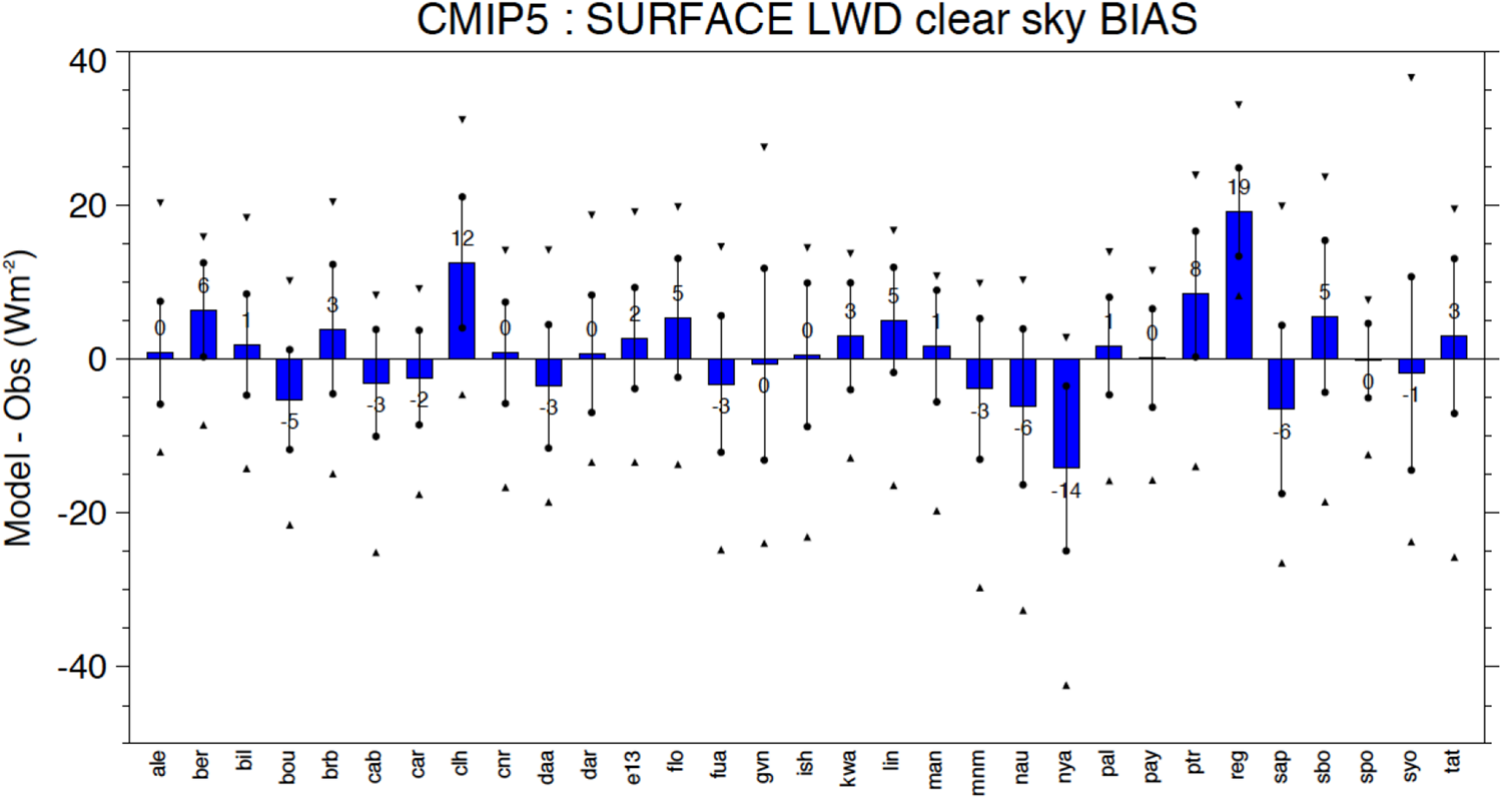
Multi-model mean biases (model-observations) in annual mean downward surface clear-sky longwave radiation at 31 different BSRN sites (blue bars). The spread of individual model biases is further indicated with a vertical line covering ± one standard deviation, and with the maximum positive and negative model biases given as triangle symbols. Station abbreviations explained in Table 1. Units Wm^−2^

The multi-model mean biases in downward surface longwave clear-sky radiation at each of the 31 BSRN sites are shown in Fig. 11 as blue bars in terms of their climatological annual mean biases. As in Fig. 5, the spread of model-calculated values at the single sites is further indicated by vertical lines covering ± one standard deviation and by the maximum positive and negative  model biases given as triangle symbols. At two-thirds of the stations, the multi-model mean bias is within 3 Wm^−2^ (blue bars in Fig. 11). The mean and median of the multi-model mean biases over all sites correspond to 1.1 and 0.9 Wm^−2^, respectively. This is remarkable given the amount of processing that went into the generation of both model-simulated and reference data.

Note that there are indications, that the official scale for longwave radiation issued by the World Meteorological Organization (WMO) based on the World Infrared Standard Group (WISG) might somewhat underestimate the downward longwave radiation measured by the pyrgeometers, and may need to be recalibrated in the future (Nyeki et al. 2017). This could potentially lead to surface downward and upward longwave fluxes which are higher by a few Wm^−2^. Until this issue is completely settled, which will require additional investigations to enhance the preliminary findings from so far four sites as well as the approval and adaptation by WMO and thus may take several years, the WISG scale will remain the reference for longwave measurements in the BSRN network.

**Fig. 12:**
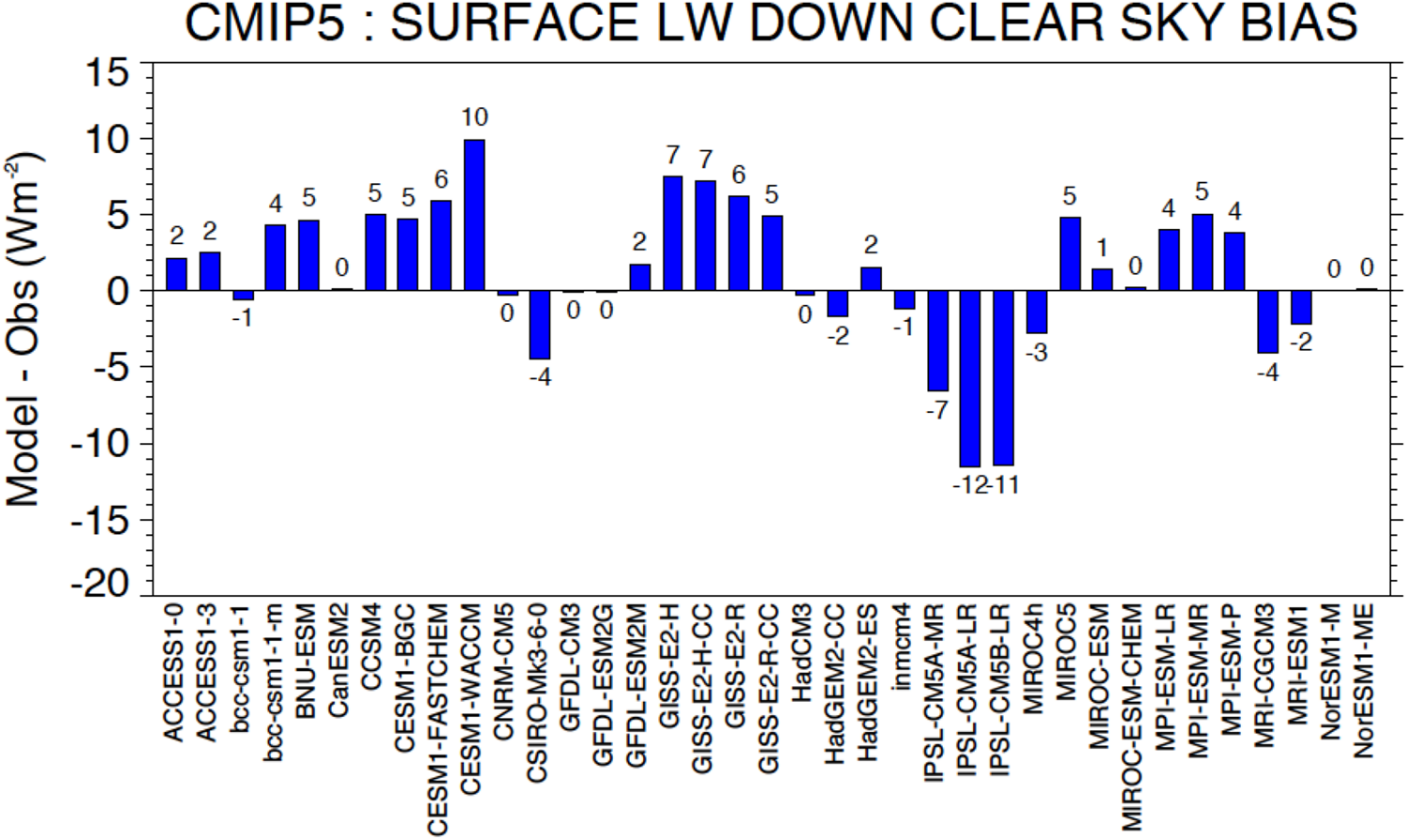
Bias (model-observations) in annual mean downward clear-sky longwave radiation at Earth’s surface calculated in 37 CMIP5 models averaged over 31 BSRN sites. Units Wm^−2^

Overall climatological mean biases in downward surface longwave clear-sky radiation of the 37 individual CMIP5 models obtained by averaging the biases over all sites are shown in Fig. 12. A large spread in the overall biases can be seen amongst the models, ranging from + 10 to − 12 Wm^−2^. As with the shortwave biases presented in Fig. 6, this large spread is not surprising, considering the substantial differences amongst the global mean downward surface longwave clear-sky radiation of the various models shown in Fig. 9 (upper panel). The majority of the models (23 out of 37) overall overestimates the BSRN reference climatologies, whereas also strong underestimations in some of the models are evident. However, there are also numerous models that show hardly any overall biases in downward surface longwave clear-sky radiation, with 11 models having an overall bias of one Wm^−2^ or less. The mean and median biases of all models only amount to 1.1 and 1.4 Wm^−2^, respectively.

### Best estimate for the global mean clear-sky downward surface longwave radiation

**Fig. 13:**
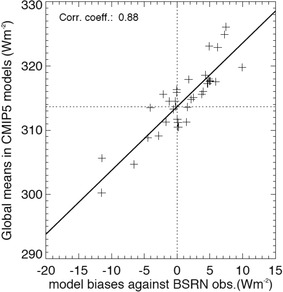
Global annual mean surface downward longwave clear-sky radiation of 37 CMIP5 models (given as crosses) on the vertical axis versus their respective overall biases compared to 31 BSRN references on the horizontal axis. A “best estimate” for the downward surface longwave clear-sky radiation is inferred at the intersect between the linear regression line and the zero bias line. Units Wm^−2^

As previously for the shortwave, we use in the following also the above longwave model biases to infer a best estimate of the global mean downward longwave clear-sky radiation at the Earth’s surface. This is again achieved by relating the model biases to their respective global mean numbers, but now for the longwave rather than the shortwave downward surface clear-sky radiation. This is shown in Fig. 13, where for each model its overall bias in the climatological annual downward surface longwave clear-sky radiation (as given in Fig. 12) is plotted against its corresponding global annual mean value (as given in Fig. 9 upper panel). As for the corresponding shortwave analysis, also in the longwave a tight relation between the two quantities can be noted, with a correlation coefficient of 0.88. The stronger the overestimation (underestimation) of the downward surface longwave clear-sky radiation at the BSRN sites in a model, generally also the higher (lower) its corresponding global mean value. Again we then infer a “best estimate” for the global mean downward surface longwave clear-sky radiation from the linear regression at the intersect where the bias against the surface observations becomes zero (dashed lines in Fig. 13). Thereby we obtain a value of 313.7 (± 0.9) Wm^−2^ (2σ uncertainty in the parentheses). Again the choice of the regression method (y-least squares and orthogonal regression) did not impact this value significantly (difference less than 0.2 Wm^−2^). We consider therefore a value around 314 Wm^−2^ as best estimate for the global mean downward surface longwave clear-sky radiation.

The very same value of 314 Wm^−2^ for this component was also obtained from the completely independent satellite-derived product of CERES EBAF-surface (Kato et al. 2013). This increases confidence in the magnitude of this quantity derived here.

Similar to the shortwave best estimate in Sect. 4.3, we also enlarge the above 2σ regression uncertainty range for the best estimate of the global mean downward surface longwave clear-sky radiation to accept all corresponding global means calculated by GCMs with marginal (< 1 Wm^−2^) biases shown in Fig. 13. Thereby we obtain a conservative uncertainty range from 311 to 317 Wm^−2^ (rounded), which is indicated in Fig. 1.

## The global energy balance under cloud-free conditions

Our best estimates of the global mean downward surface shortwave and longwave clear-sky radiation of 247 and 314 Wm^−2^, respectively, as derived in the above sections can then be combined with additional estimates of the TOA clear-sky fluxes and surface radiative properties, to allow for a quantitative diagram of the global mean energy balance under cloud-free conditions (Fig. 1). Note that the clear-sky flux magnitudes in this diagram are representative for an atmospheric structure corresponding to all-sky conditions but with clouds removed. This view thus corresponds to the climate model-type representation of clear-sky fluxes (Method II according to Cess and Potter 1987), and enables a direct comparison with the clear-sky budgets obtained from GCM output. It further allows the isolation and quantification of the effects of clouds on the global mean Earth radiation budget, by comparing the all-sky fluxes with the clear-sky fluxes merely obtained by removing the clouds, but with otherwise identical atmospheric conditions.

**Fig. 14:**
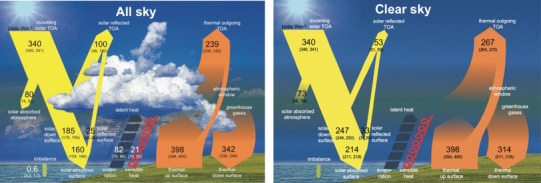
Diagrams of the global mean energy balance of the Earth under all-sky (left) and clear-sky conditions (right), representing present day climate at the beginning of the 21th century. All-sky diagram from Wild et al. (2015). Units Wm^−2^

The currently most accurate estimates for the global mean clear-sky fluxes at the TOA as presented in Fig. 1 can be obtained from the CERES EBAF dataset. We use here data from its latest Edition 4.0 (Loeb et al. 2018), where the global annual mean estimates for the reflected shortwave clear-sky and outgoing longwave clear-sky fluxes amount to 53 and 268 Wm^−2^, respectively. These values, however, are aggregated only from cloud-free scenes, and thus, similarly to the BSRN shortwave clear-sky climatologies discussed previously, may slightly differ from the computation of clear-sky TOA fluxes in climate models (c.f. Sect. 4.2). The effect of sampling over all types of atmospheric conditions as in climate models, rather than only over truly cloud-free conditions in the calculation of the global mean clear-sky fluxes have been estimated by Kato et al. (2013) using the CERES EBAF framework. For the global mean shortwave reflected and outgoing longwave radiation at the TOA, Kato et al. (2013) estimate this sampling effect at 0.24 and − 1.25 Wm^−2^, respectively (their Table 2). This signifies, that considering not just the (slightly drier) atmospheres as under true clear-sky conditions, but all atmospheric conditions (with somewhat more water vapor content) in the calculation of the clear-sky fluxes, results in a slightly lower outgoing clear-sky longwave and slightly higher reflected clear-sky shortwave radiation at the TOA, as one would expect. To keep the clear-sky energy balance diagram consistent with the GCM definition of clear-sky fluxes (Method II), we therefore slightly adjusted the CERES TOA global mean values for Fig. 1 with the abovementioned corrections of 0.24 and − 1.25 Wm^−2^ in the shortwave and longwave, respectively. With these corrections, rounded to integers, the global mean clear-sky TOA shortwave reflected radiation remains at 53 Wm^−2^, while the outgoing longwave radiation is slightly reduced to 267 Wm^−2^ (Fig. 1). The associated uncertainties of these estimates amount to ± 2 and ± 3 Wm^−2^, respectively (Fig. 1) (Loeb et al. 2009). Combined with a global mean TOA insolation of 340 Wm^−2^ (solar constant divided by four to obtain the average TOA insolation on a square meter on the Earth’s sphere), the total amount of shortwave absorption under cloud-free conditions becomes 287 Wm^−2^. Note that the cloud-free energy balance in the diagram shown in Fig. 1 is not the balance that Earth would achieve in equilibrium when no clouds could form. As mentioned above the diagram is rather representative for the GCM-type of clear-sky fluxes determined by removing the clouds but otherwise retaining the entire atmospheric structure. As a consequence, unlike in the all-sky case, the TOA budget is far from being balanced between shortwave clear-sky absorption (287 Wm^−2^) and longwave clear-sky emission (267 Wm^−2^). In the all-sky budget (Fig. 14 left, reproduced from Wild et al. 2015) the difference between these two quantities is largely remediated by the global net cloud radiative effect.

In our representation of the clear-sky energy balance we further retain the same radiative properties for the surface emission and surface albedo as in the discussion of the all-sky budget in Wild et al. (2015), to be consistent. Using thus the global surface albedo of 13.5% from Wild et al. (2015) and the best estimate of 247 Wm^−2^ for the global mean clear-sky shortwave downward radiation (Sect. 4.3), the global mean clear-sky surface shortwave absorption and reflection become 214 and 33 Wm^−2^, respectively (Fig. 1). A detailed description of the derivation of the surface albedo value can be found in Wild et al. (2015). Strictly speaking, the surface albedo under clear-skies slightly differs from the one under all-sky conditions, because the incident radiation under clear-skies is more directional particularly over ocean. According to the dataset of CERES EBAF surface, this effect is, however, small on a global mean basis, at 0.3% (Kato et al. 2018, as can be derived from their Table 5). This corresponds to a change in surface shortwave reflection of only 0.7 Wm^−2^, so that the magnitude of this flux rounded to integer remains at 33 Wm^−2^ as given in Fig. 1.

The amount of shortwave radiation absorbed within the cloud-free atmosphere can then be determined as residual between the cloud-free absorption of shortwave radiation in the total (TOA) climate system (287 Wm^−2^) and at the surface (214 Wm^−2^), and thus amounts to 73 Wm^−2^. The 73 Wm^−2^ of atmospheric shortwave clear-sky absorption closely match our previous estimates of this quantity which we obtained more than a decade ago based on older models and their biases against much fewer direct clear-sky observation (Wild et al. 2006). Two decades ago we estimated the global mean clear-sky atmospheric absorption similarly at 72 Wm^−2^, not derived from clear-sky climatologies as they were not available at the time, but from a stand-alone evaluation of the radiation codes under cloud-free conditions used in the ECHAM3 and ECHAM4 climate models (Wild et al. 1998). Also, from the completely independently derived CERES-EBAF Ed.4. satellite-derived dataset, the very same global mean atmospheric shortwave clear-sky absorption of 73 Wm^−2^ has been determined (Kato et al. 2018) as in the present study. Further, in another independent approach, Kim and Ramanathan (2008) obtained a closely matching estimate of 72 Wm^−2^ for this quantity, by integrating global satellite-derived data sets for aerosols, water vapor and total ozone with a Monte Carlo Aerosol-Cloud-Radiation (MACR) model.

This indicates that the estimate of the global mean shortwave absorption in the cloud-free atmosphere, somewhat above 70 Wm^−2^, seems to be fairly robust. Thus, about 21% of the incoming shortwave radiation at the TOA, or about 30% of the total amount of shortwave radiation absorbed by Earth may be absorbed in the cloud-free atmosphere.

The 73 Wm^−2^ derived here for the global mean clear-sky shortwave atmospheric absorption are distinctly higher than some of the earlier estimates, and many of the simulated values of this quantity in GCMs, particularly in older models. This fits to the evidence for a lack of shortwave atmospheric absorption in many of the GCM radiation codes compared to line-by-line reference codes in radiation code intercomparison projects spanning more than two decades (Fouquart et al. 1991; Barker et al. 2003; Oreopoulos et al. 2012). The situation in GCMs has somewhat improved over the years. While the global mean clear-sky shortwave atmospheric absorption in some of the GCMs of the early 1990s did not even reach 60 Wm^−2^ (Wild et al. 1998), this quantity was gradually enhanced in subsequent GCM generations, to 67 and 69 Wm^−2^ in terms of multi-model means in the AMIPII and CMIP3 model intercomparison projects, respectively (Wild et al. 2006), and finally to 71 Wm^−2^ in the CMIP5 multi-model mean (Table 1). This development is in line with the notion of an overall improvement in the performance of more recent GCM radiation codes in the context of the Continual Intercomparison of Radiation Codes (CIRC) compared to earlier assessments (Oreopoulos et al. 2012). Increasing water vapor absorption, due to the improved description of the shortwave continuum absorption in the near-infrared windows and revised spectroscopic absorption coefficients, may favour this development (Radel et al. 2015; Paynter and Ramaswamy 2012, 2014; Kim and Ramanathan 2008). A more accurate description of the aerosol radiative properties in the GCM radiative transfer calculations may further contribute to the enhanced shortwave absorption (Ackerman et al. 2003; Kim and Ramanathan 2008). Note that now about half of the CMIP5 models calculate a clear-sky atmospheric absorption that exceeds 70 Wm^−2^ (Fig. 2 middle panel), and are thus in close agreement with the best estimate supported here. Yet there are still a number of CMIP5 models with a substantially lower clear-sky atmospheric absorption (Fig. 2 middle panel). This low absorption in some of these models may rather point to an inadequate implementation of known radiation physics in their radiation codes than to remaining genuine uncertainties in the representation of aerosols and the water vapor continuum.

In the longwave part of the cloud-free global energy balance diagram in Fig. 1, the surface fluxes consist of the downward longwave clear-sky radiation of 314 Wm^−2^ (Sect. 5.3), and the upward longwave clear-sky surface radiation of 398 Wm^−2^. This latter flux remains essentially the same as in the all-sky diagram (Fig. 14 left), since in the clear-sky we keep the surface and atmospheric properties identical to the all-sky except for the clouds as pointed out earlier, and the emission in the clear-sky thus comes from the surface with the same temperature as the all-sky. For a discussion of the justification of the 398 Wm^−2^ for the all/clear-sky longwave surface upward radiation the reader is referred to Wild et al. (2015). To be precise, there would still be a slight difference between the clear-sky and all-sky surface upward longwave radiation even under otherwise identical surface and atmospheric conditions. This is due to the fact the surface upward longwave flux contains not only the longwave emission from the Earth’s surface, but added to this also a small contribution of the upward reflected fraction of downward longwave radiation. This reflection is neglected when the Earth’s surface is assumed to be a perfect blackbody in the longwave, which then absorbs all incident longwave radiation. In reality the Earth’s surface is globally indeed fairly close to a blackbody in the longwave, yet not perfectly, allowing for a few percent of the downward longwave fluxes to be reflected at the surface. However, since the all-sky and clear-sky downward longwave fluxes overall differ globally by only about 28 Wm^−2^ (Table 2 and following Section below), the difference in the surface reflected longwave flux under clear- and all-sky conditions is less than 1 Wm^−2^. This slight difference is thus neglected in Figs. 1 and 14, which display identical surface upward longwave fluxes under clear-sky and all-sky conditions. With a surface upward and downward clear-sky longwave radiation of 398 and 314 Wm^−2^, this results in a best estimate for the surface net longwave clear-sky radiation of − 84 Wm^−2^. This allows then also the estimation of the atmospheric clear-sky longwave net radiation (longwave atmospheric clear-sky divergence) at − 183 Wm^−2^, calculated as a residual between the clear-sky outgoing and surface net longwave radiation of − 267 and − 84 Wm^−2^, respectively. The corresponding value for the atmospheric clear-sky longwave net radiation as derived in CERES-EBAF according to Kato et al. (2013) amounts to − 182 Wm^−2^. Thus, as with the shortwave clear-sky atmospheric absorption, also our estimates for its longwave counterpart closely agree with the corresponding independent CERES-EBAF estimates.

## The global cloud radiative effects

**Fig. 15:**
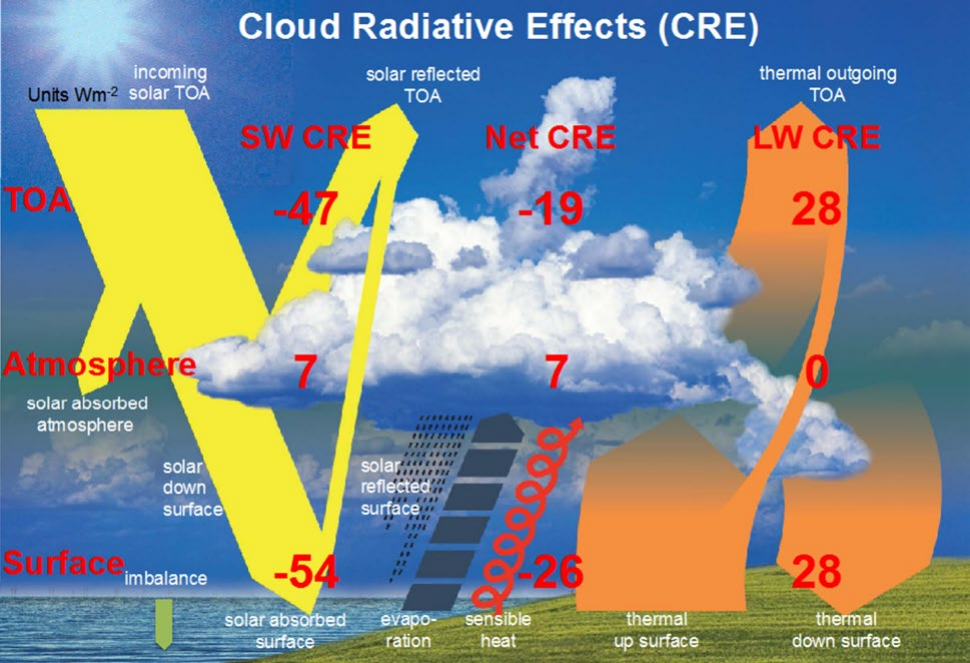
Illustration of the magnitudes of the global mean shortwave, longwave and net (shortwave + longwave) cloud radiative effects (CRE) at the Top-of-Atmosphere (TOA), within the atmosphere and at the Earth’s surface, determined as differences between the respective all-sky and clear-sky radiation budgets presented in Fig. 14. Units Wm^−2^

The clear-sky radiation balance derived in the previous section can then be used as a reference state to isolate and quantify the overall radiative effects of clouds, through a comparison with the corresponding all-sky radiation balance. Figure 14 contrasts the two radiation budgets, including clouds (left, as estimated in Wild et al. 2015) and excluding clouds (right, as estimated in the present study). This not only allows the estimation of the overall effects of clouds on the global mean radiation budget at the TOA, but also within the atmosphere and at the Earth’ surface, as given in Fig. 15.

The TOA shortwave absorption under all-sky and clear-sky conditions as determined from CERES-EBAF (Loeb et al. 2018), at 287 and 240 Wm^−2^, respectively, differs by 47 Wm^−2^. This suggests that the overall effect of clouds is to reduce the absorption of shortwave radiation in the climate system by − 47 Wm^−2^ (TOA shortwave cloud radiative effect). Since the cloud radiative effect is defined as the difference between the all-sky and clear-sky fluxes, and since this indicates a cooling of the climate system, this value obtains a negative sign (Table 2; Fig. 15). Accordingly, the longwave cloud radiative effect at the TOA, as the difference between the outgoing longwave radiation under all-sky and clear-sky conditions determined from CERES-EBAF Edition 4.0, becomes positive at 28 Wm^−2^, since this states an energy gain for the climate system (Table 2; Fig. 15). The net (shortwave and longwave combined) cloud radiative effect at the TOA then results in an overall energy loss of − 19 Wm^−2^ (Table 2; Fig. 15).

In addition to these widely published TOA cloud radiative effects, our study further allows the estimation of the global mean shortwave, longwave and net cloud radiative effects within the atmosphere and at the surface in a similar way, based on the respective all-sky and clear-sky estimates given in Fig. 14. At the surface the shading effects of clouds are estimated to reduce the downward shortwave radiation globally from 247 to 185 Wm^−2^, thus by − 62 Wm^−2^. In terms of absorbed (net) shortwave radiation at the Earth surface, the reduction by clouds amounts to − 54 Wm^−2^ globally (from 214 to 160 Wm^−2^), which corresponds to the net surface shortwave cloud radiative effect (Table 2; Fig. 15). On the other hand, clouds are estimated to enhance the global mean surface downward longwave radiation by 28 Wm^−2^, from 314 to 342 Wm^−2^ (Table 2; Fig. 15). The same amount applies also to the net surface longwave cloud radiative effect (i.e. the cloud effect on the net longwave balance at the surface), since the all-sky and clear-sky upward longwave radiation is nearly identical within this framework (see discussion above). The surface net cloud radiative effect, defined as the sum of the surface net shortwave and longwave cloud effects, thus amounts then to − 26 Wm^−2^ globally (Table 2).

In the atmosphere, the presence of clouds is estimated to enhance the shortwave absorption by 7 Wm^−2^ from 73 Wm^−2^ (Fig. 14 right), to 80 Wm^−2^ (Fig. 14 left). This is slightly higher than corresponding estimates obtained from most climate models and the CERES EBAF datasets, at 5 Wm^−2^ between 60°N and 60°S (Hakuba et al. 2016), and 4.1 Wm^−2^ globally (Kato et al. 2018).

Since the cloud radiative effect on the longwave budget of the atmosphere is virtually zero (as a residual of surface and TOA longwave cloud radiative effects of 28 Wm^−2^ each), the atmospheric net cloud radiative effect also remains at 7 Wm^−2^ (Table 2; Fig. 15).

**Fig. 16:**
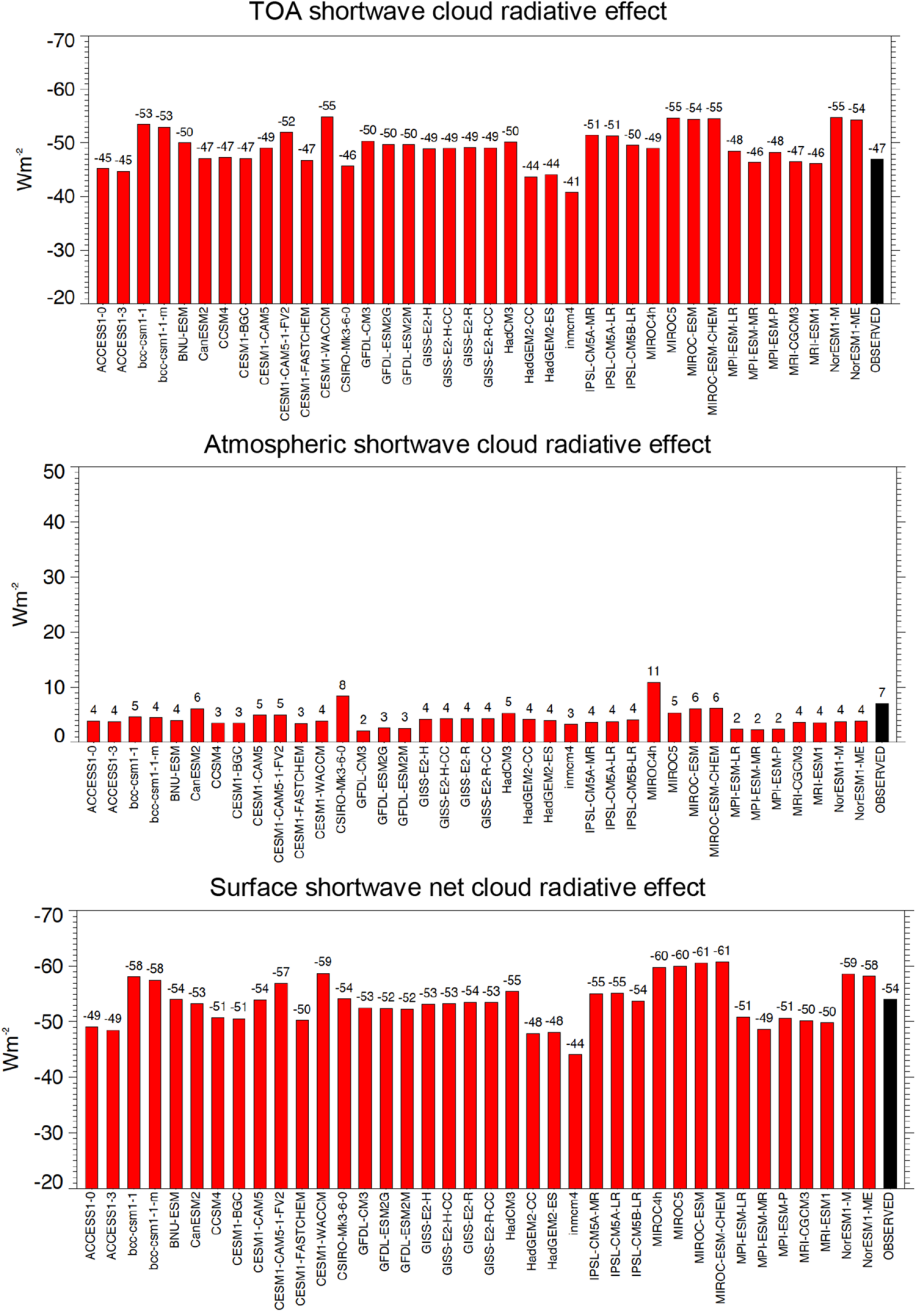
Global mean shortwave cloud radiative effects at the TOA (upper panel), within the atmosphere (middle panel) and at the surface (lower panel), defined as differences between the respective all-sky and clear-sky radiation budgets in individual CMIP5 models (red bars) and as derived as best estimate in the present study (black bars)

**Fig. 17:**
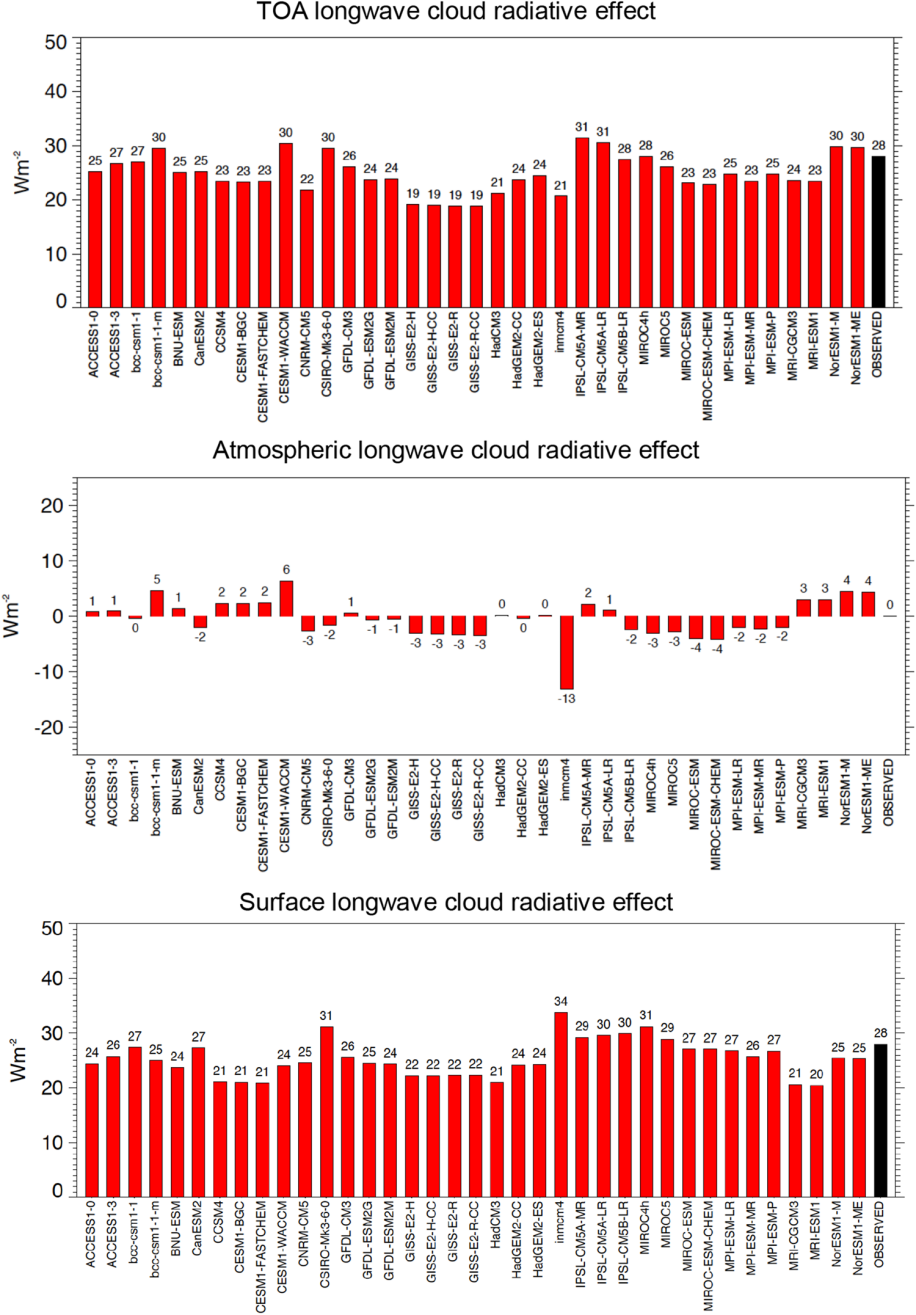
Global mean longwave cloud radiative effects at the TOA (upper panel), within the atmosphere (middle panel) and at the surface (lower panel), defined as differences between the respective all-sky and clear-sky radiation budgets in individual CMIP5 models (red bars) and as derived as best estimate in the present study (black bars)

**Fig. 18:**
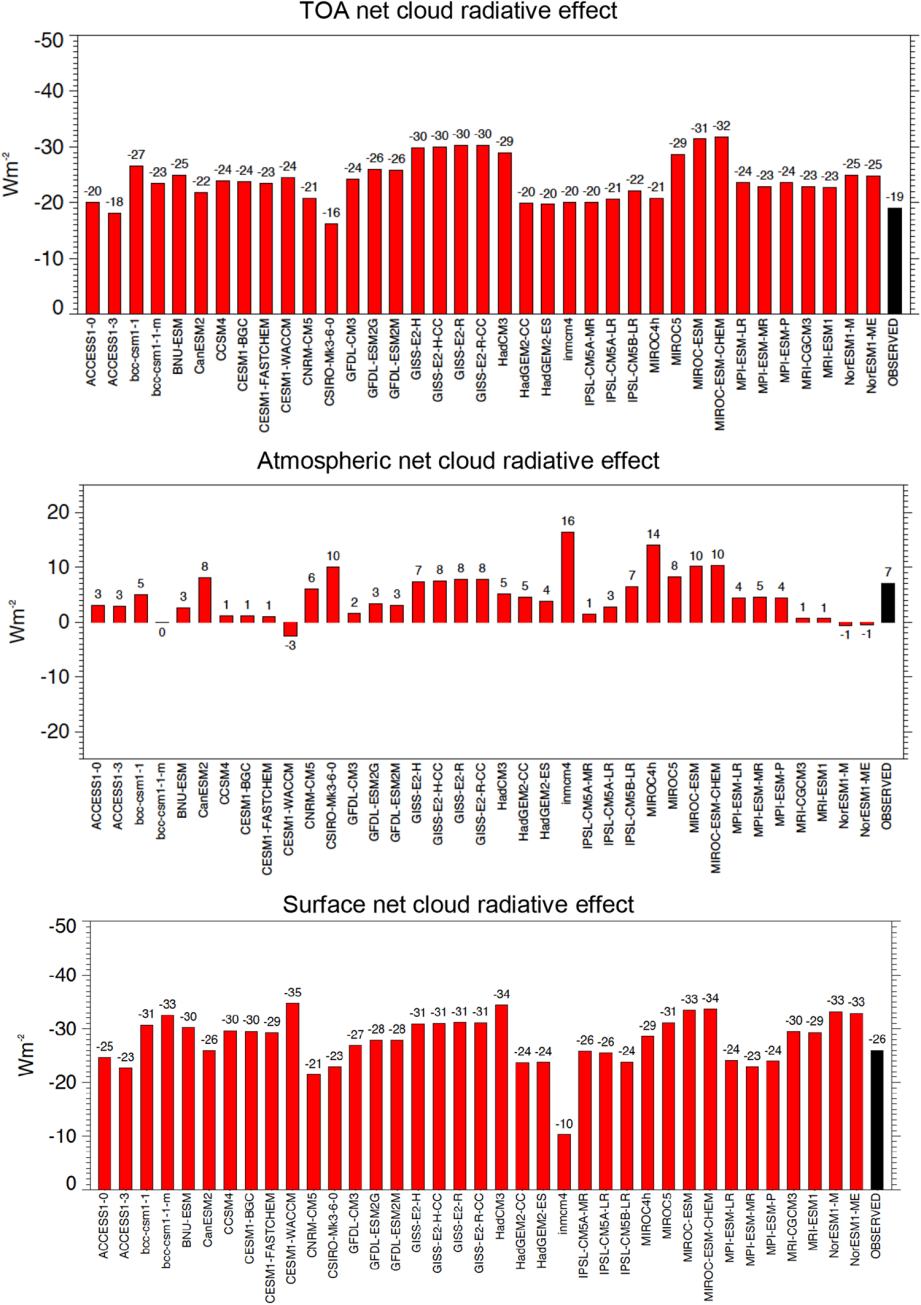
Global mean net (shortwave + longwave) cloud radiative effects at the TOA (upper panel), within the atmosphere (middle panel) and at the surface (lower panel), defined as differences between the respective all-sky and clear-sky radiation budgets in individual CMIP5 models (red bars) and as derived as best estimate in the present study (black bars)

These estimates for the surface, atmospheric and TOA global mean shortwave, longwave and net cloud radiative effects inferred here and illustrated in Fig. 15 can then be compared to the respective values determined in the various CMIP5 models (multi-model means in Table 2, individual models in Figs. 16, 17, 18). In the multi-model global mean, both shortwave and longwave cloud radiative effects agree within a few Wm^−2^ with the reference estimates derived here (Table 2). Global mean cloud radiative effects of individual models, however, vary substantially, with largest spreads and standard deviations occurring in the simulated surface estimates (Table 2; Figs. 16, 17, 18). This leads in some of the models to substantial deviations compared to the reference values developed here, in some cases exceeding 10 Wm^−2^ on a global mean basis (Figs. 16, 17, 18). The deviations of the model-calculated surface cloud radiative effects from the reference values, together with the biases in their associated clear-sky fluxes, may give an indication for each individual model to what extent its cloud-free atmosphere or its clouds contribute to the overall biases noted in the simulated (all-sky) surface fluxes in previous studies.

## Summary and conclusions

The global energy balance quantifies the distribution of radiative energy in the climate system. In previous studies we derived estimates for the magnitudes of the components of the “all-sky” global mean energy balance, taking into account the information provided by direct observations from surface and space as well as climate model estimates (Wild et al. 2013, 2015). In the present study we made an attempt to derive complementary estimates for the global mean energy fluxes under cloud-free (“clear-sky”) conditions. While reference values for the energy fluxes in and out of the climate system under cloud-free conditions at the TOA are now available with high accuracy from satellite-based measurements (CERES EBAF), corresponding estimates for the atmospheric and surface clear-sky budgets are less straightforward to obtain. Yet such reference values are needed, since climate models still show considerable spreads in their simulated clear-sky budgets, particularly at the Earth surface. As outlined in this study, these model spreads amount already in the global mean to as much as 16 and 24 Wm^−2^ for the clear-sky surface downward shortwave and longwave radiation, respectively, which may further amplify on regional, seasonal and diurnal scales. We thus made an attempt to better constrain these fluxes using newly-derived clear-sky reference climatologies obtained at more than 50 worldwide distributed anchor sites from BSRN. The assessment has been slightly complicated by the fact that the monthly shortwave clear-sky BSRN reference climatologies are derived from measurements under truly cloud-free conditions, whereas the GCMs clear-sky fluxes are calculated continuously at every time-step solely by removing the clouds, yet otherwise retaining the prevailing atmospheric composition associated with the cloudy conditions. The quantitative effects of these different clear-sky definitions were estimated by comparing in multi-century GCM control simulations the clear-sky irradiances in months with lowest cloud amounts to their corresponding climatological mean clear-sky irradiances. The effects of the different clear-sky definitions between models and observations, estimated at 2 Wm^−2^ on average, were individually determined for each model and month and adjusted accordingly for a proper comparison with the reference climatologies.

The subsequent assessment of the CMIP5 clear-sky surface flux climatologies at the BSRN sites identified a number of models with overall biases of no more than 1–2 Wm^−2^ in their shortwave and/or longwave downward flux climatologies. However there remain also a number of models with substantially larger overall flux biases, indicative of strong overestimations of the downward shortwave fluxes, as well as both overestimations and underestimations of the downward longwave fluxes, with systematic biases up to more than 10 Wm^−2^ when averaged over all BSRN sites.

To derive best estimates for the global mean clear-sky radiative fluxes at the Earth’s surface we then applied the approach presented in Wild et al. (2013, 2015) taking into account the bias structure of the 38 CMIP5 models with respect to the BSRN sites. The clear-sky radiative flux biases in the various models have thereby been linearly related to their respective global means. From the linear regression we inferred a best estimate (with zero bias against the surface observations) of 247 Wm^−2^ for the global mean downward shortwave radiation at the surface under cloud-free conditions, and a corresponding surface absorption of 214 Wm^−2^, assuming a global mean surface albedo of 13.5%. Combined with a best estimate for the global net influx of shortwave radiation at the TOA under cloud-free skies from CERES-EBAF of 287 Wm^−2^, this leaves 73 Wm^−2^ of shortwave radiation absorbed globally in the cloud-free atmosphere. This estimate nearly matches the completely independent estimates obtained from the state of the art satellite-retrieved dataset CERES-EBAF (Kato et al. 2013), as well as from a Monte Carlo Aerosol-Cloud-Radiation (MACR) model assimilated with satellite-derived input (Kim and Ramanathan 2008).

For the global mean downward longwave clear-sky radiation, a best estimate of 314 Wm^−2^ was obtained, based on a regression between the GCM flux biases in downward longwave clear-sky radiation with respect to the BSRN references and their corresponding global means. Also this value matches the corresponding one from the independent CERES-EBAF satellite-retrieved dataset (Kato et al. 2013).

The close coincidence in the global magnitudes of key quantities describing the energy flows within the cloud-free climate system obtained in multiple independent approaches (based on surface observations and climate models as presented here on the one hand, and on state of the art satellite-derived products on the other hand) increases confidence in the robustness of the derived magnitudes of these global numbers.

Accurate knowledge of the global radiation budget of the cloud-free atmosphere is a prerequisite to quantify the global cloud radiative effects, through a comparison with consistently derived “all-sky” global radiation budget estimates. Quantitative estimates of the global cloud radiative effects at the TOA, within the atmosphere and at the surface have thus been derived in the present study, which may also be of use in climate model assessments and tuning.

While the focus in the present study was on a first-order quantification of the overall (global long-term mean) clear-sky budgets and cloud radiative effects, future studies should expand to assess these quantities in detail in their spatial as well as temporal variations.
